# Deltamethrin resistance in the salmon louse, *Lepeophtheirus salmonis* (Krøyer): Maternal inheritance and reduced apoptosis

**DOI:** 10.1038/s41598-018-26420-6

**Published:** 2018-05-31

**Authors:** Marit Jørgensen Bakke, Celia Agusti, Jo Christiansen Bruusgaard, Arvind Y. M. Sundaram, Tor Einar Horsberg

**Affiliations:** 1 Norwegian University of Life Science, Faculty of Veterinary Medicine, Sea Lice Research Centre, Oslo, Norway; 2 Department of Health Sciences, Kristiania University College, Oslo, Norway; 3 Department of Biosciences, University of Oslo, Oslo, Norway; 4 Department of Medical Genetics, Oslo University Hospital and University of Oslo, Oslo, Norway

## Abstract

Resistance towards deltamethrin (DMT) in the crustacean ectoparasite *Lepeophtheirus salmonis* (Caligidae) is a problem on fish farms lining the North Atlantic Ocean. Two Norwegian strains with different susceptibility towards DMT were crossed in the parental generation (P0), females from a sensitive strain were crossed with males from a resistant strain and vice versa. Individual susceptibility towards DMT was assessed in the second filial generation (F2). DMT resistance was only found in F2 descendants when the P0 females were from the resistant strain, pointing to maternal inheritance. Since maternal inheritance might be linked to the mitochondrial (mt) genome, the nucleotide sequences and the gene expressions of mt-genes were analysed. Twenty non-synonymous single nucleotide polymorphisms (SNPs) were identified in mt-transcripts from resistant F2 parasites, including SNPs in two cytochrome C oxidase subunits (*COX1* and *COX3*) and two subunits of the NADH dehydrogenase complex (*ND1* and *ND5*) previously linked to DMT resistance in the salmon louse. Differential expression analysis between the sensitive and resistant strain revealed strain effect in seven out of twelve mt-genes. The current study also show that DNA fragmentation (indicating apoptosis) was affected by DMT exposure in skeletal muscle tissue and that resistant parasites undergo less apoptosis than sensitive parasites.

## Introduction

The use of pesticides is important to combat arthropod pests and parasites on terrestrial animals and crops (insects and arachnids), and in the aquaculture industry (crustacean parasites). The same pesticides are also used to reduce the number of vectors for human diseases, such as mosquito species that carry malaria parasites, zika virus and dengue virus. One of the most commonly used classes of pesticides are the pyrethroids, which includes deltamethrin (DMT). Sea lice (Caligidae) are crustacean ectoparasites on marine fish that can have serious consequences for farmed fish. The caligid copepod *Lepeophtheirus salmonis* (the salmon louse) is a major problem in salmonid fish farming in Norway and other countries lining the North Atlantic Ocean, such as Scotland, Ireland, the Faroe Islands and Canada^1^. Although non-pharmaceutical measures are commonly taken, chemical treatments are still necessary to keep lice-counts under regulatory limits (0.5 adult females per fish in Norway). Resistance towards the compounds is widespread^2–4^. In Chile, the caligid copepod *Caligus rogercresseyi* constitute a similar problem in the salmonid marine aquaculture as *L*. *salmonis* does on the Northern hemisphere and the same pesticides are used in the combat against them.

Generally, it is believed that pyrethroids bind to the voltage gated sodium channel (VGSC) in nerves and causes a prolonged opening of the channels^5^. A continuous excitation results in paralysis and ultimately death of the organism. In insects and arachnids, specific mutations in the gene coding for the VGSC (*Na*_*V*_) protect the animals from pyrethroid toxicity e.g.^6,7^. The mode of action in crustaceans is assumed similar to other arthropod groups, although poorly investigated. Attempts to identify mutations in the *Na*_*V*_ linked to pyrethroid resistance in *L*. *slamonis* have failed^8^. An overrepresentation of a non-synonymous single nucleotide polymorphism (SNP) in *Na*_*V*_, in a pyrethroid resistant *L*. *salmonis* population has been reported^9^ but could not be found in resistant individuals from a different population^8^.

Maternal inheritance of resistance in arthropods is rare, previously only described in the two-spotted spider mite (*Tetranychus urticae*) against bifenazate^10^ and its cross-resistance to acequinocyl^11^. Nevertheless, two studies suggest that DMT resistance in the salmon louse is maternally inherited^12,13^. In both crossing studies, only resistant females, and not resistant males, produced resistant offspring. Furthermore, both studies identified SNPs between DMT-susceptible and DMT-tolerant parasites in the mitochondrial DNA (mt-DNA). Although natural occurring polymorphisms are expected, especially in the mt-genome^14^, the distinct differences between susceptible and resistant lice from both Scotland and Norway suggest that these SNPs are related to DMT susceptibility. The genes encoded in the mt-DNA are coding for proteins involved in the electron transport chain (ETC), which is the main source for ATP production in the cells. How changes in the ETC complexes may compensate for DMT exposure is not clear. However, the ATP levels have been described to be higher in resistant lice compared to susceptible lice^13^, further implying that alterations in the ETC are in some way involved in the protection from detrimental DMT toxicity.

Deltamethrin has been demonstrated to induce controlled cell death, apoptosis, in murine thymocytes and human neuroblastoma cell cultures^15,16^. Apoptosis can be induced by low levels of ATP that lead to disruption of the mitochondria membrane potential between the intermembrane space and the cytosol, resulting in leakage of cytochrome C (cytC) into the cytosol. Further downstream effects are caspase3 activation and ultimately DNA fragmentation.

The main aim of this study was to identify differences in the mt-DNA between resistant and susceptible lice (*L*. *salmonis*), both in terms of SNPs and expression levels. A fully susceptible strain and a pyrethroid resistant strain, both originating from Norwegian fish farms, were used in this pursuit, in addition to the second filial generation (F2) after crossing them. An additional aim was to investigate whether a link between DMT exposure and apoptosis could be established, and to see if resistant and susceptible lice respond differently.

## Results

### Crossing experiment and bioassays

**Figure 1:**
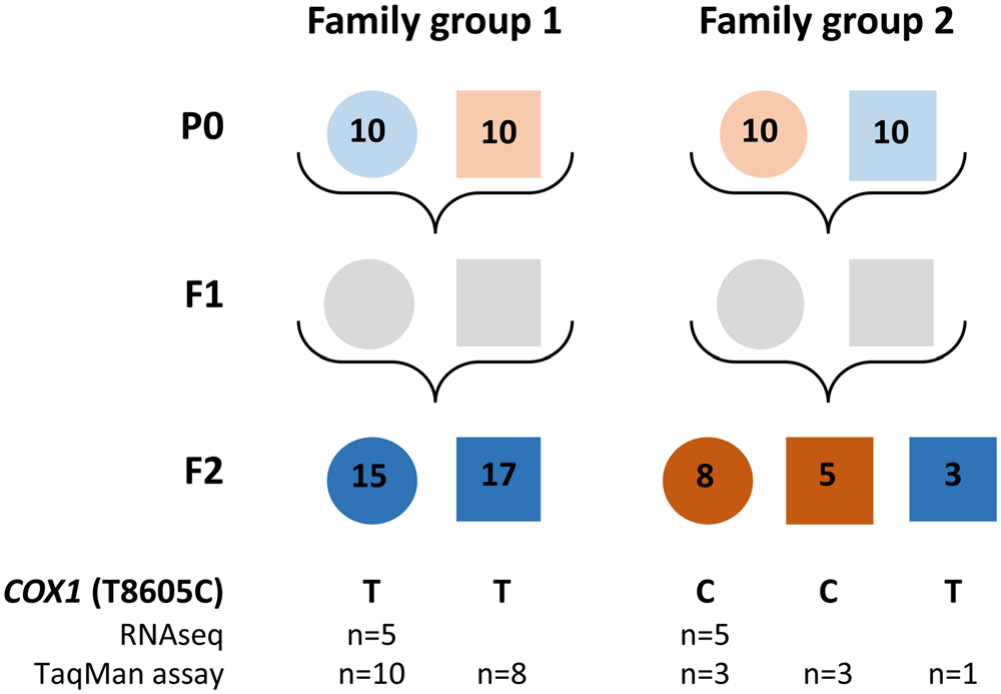
Crossing experiment with one fully sensitive *L*. *salmonis* strain (Ls A, light blue) and one DMT-tolerant strain (Ls V, light brown), evaluated on population level (circles = females, squares = males). The DMT susceptibility in the F1-generation was not tested (grey). In the F2-generation, parasites were characterized individually, as sensitive if they were immobilized after exposure to a low DMT concentration (0.2 µg/L) and resistant if they were unaffected after exposure to a high DMT concentration (1.0 µg/L). All F2 progenies in family group 1 were sensitive (blue). The progenies in family group 2 were mostly resistant to DMT (brown), although a few males were characterized as sensitive (blue). The *COX1 8605C* SNP always followed the resistant phenotype, whereas parasites characterized as sensitive carried the wildtype *T8605* in *COX1*. The presence of the SNP was determined after transcriptome analysis (RNAseq) or by TaqMan assay.

Two Norwegian strains with different susceptibility towards DMT were batch crossed in the parental generation (P0); Females from a sensitive strain (Ls A) were crossed with males from a resistant strain (Ls V) and vice versa, giving rise to family groups 1 and 2, respectively (Fig. 1). This was done with ten females and ten males randomly mating in each family group. In the F1 generation, the lice were allowed to grow to adults and mate naturally within their family groups to produce the F2 generation. Individual DMT susceptibility was recorded with bioassay in the F2 generation, both on preadult and adult stages (Table 1). Parasites immobilized at 0.2 µg DMT/L (24 h) were characterized as sensitive and parasites unaffected by 1.0 µg DMT/L (24 h) were characterized as resistant. All parasites in the F2-generation of family group 1, where the P0 females were from the sensitive Ls A strain, were immobilized at both 0.2 µg/L and 1 µg/L DMT-concentrations and thus characterized as sensitive. In family group 2, where the P0 females were from the resistant Ls V strain, none of the females were immobilized, whereas a low fraction of the males were immobilized (9% and 17% for preadults and adults, respectively) at the lowest concentration (0.2 µg DMT/L). At the highest concentration (1 µg DMT/L), there was partial but not complete immobilization of the parasites from family group 2; 28% of the females and 15% of the males were characterized as resistant as described above.

**Table 1:** Immobilization (%) in the F2 generation after crossing between a DMT-sensitive salmon louse strain (Ls A) and a DMT-resistant salmon louse strain (Ls V).

		0 µg DMT/L		0.2 µg DMT/L		1.0 µg DMT/L		(n)	
		♀	♂	♀	♂	♀	♂	♀	♂
Family group 1	preadult	0%	0%	100%	100%	100%	100%	36	39
Family group 2	preadult	10%	0%	0%	9%	67%	86%	87	66
Family group 1	adult	0%	0%	100%	100%	100%	100%	22	31
Family group 2	adult	0%	0%	0%	17%	72%	85%	43	37

Female Ls A and male Ls V (family group 1) or female Ls V and male Ls A (family group 2) were batch crossed in the P0 generation (see Fig. 1).

♀ = female and ♂ = male.

### SNP analysis

From the individually characterized parasites in the F2-generation, sensitive and resistant parasites were sampled and five from each group were subjected to transcriptome sequencing (RNAseq). The consensus sequences of the whole mitochondria (mt) genome derived from the transcripts aligning to the mt-genome (GenBank AY625897.1) were retrieved for each parasite individually. These mt-genome sequences from five sensitive and five resistant F2 females were then aligned to identify SNPs. No alignments to the putative ATP synthase subunit 8 gene were found.

There were 175 consistent SNPs identified in the F2-resistant lice, of which 51 were located in non-coding regions, one was located in the tRNA for the amino acid isoleucine, seven were located in rRNA coding regions, and 125 were located in the 12 mt-genes (Table 2). Twenty of the SNPs were non-synonymous, resulting in amino acid changes in the corresponding proteins, and they were found in all the F2-resistant parasites as opposed to all F2-sensitive parasites that did not carry these non-synonymous SNPs. The presence or absence of nineteen of these SNPs was used to define a genotype representing DMT resistant parasites (genotype R) and a genotype representing DMT sensitive parasites (genotype S), respectively. The non-synonymous SNP *A7391C*, resulting in Met1Ile in ND1, was considered irrelevant^17^. Four of the non-synonymous SNPs were identical to amino acid substitutions described in DMT-resistant lice from Scotland^13^, thus the presence of these amino acid changes (Gly251Ser in ND1, Leu411Ser in ND5, Leu107Ser in COX1 and Gly33Glu in COX3) will be emphasized in the following sections. A full list of SNPs and their position is given in the Supplementary Material (Table S1), where the numbering refers to the mitochondria genome published by Tjensvoll *et al*.^18^, GenBank AY625897.1.

**Table 2:** Number of total SNPs and non-synonymous SNPs derived from the F2 samples.

	No. of SNPs	No. of Non-synonymous	Amino acid changes
NC region	49		
*tRNA*(*Ile*)	1		
*rRNA*	6		
*ND1*	8	3	Met1Ile, Phe236Leu, Gly251Ser
*ND*2	20	5	Gly136Ser, Ser285Gly, Gly286Ser, Ala296Val, Phe297Leu
*ND*3	5	2	Thr32Ala, Met88Val
*ND*4	13	4	Ala161Thr, Leu323Met, Ile371Val, Ser395Lys
*ND4L*	4	0	
*ND*5	20	2	Gly28Val, Leu411Ser
*ND*6	3	1	Thr100Ala
*CytB*	7	0	
*COX1*	16	1	Leu107Ser
*COX2*	8	0	
*COX3*	10	1	Gly33Glu
*ATP6*	5	1	Val34Ala

The SNPs are located in the non-coding region, 1 tRNA coding region, 2 rRNA coding regions, and in the 12 mitochondria genes. The resulting amino acid changes from non-synonymous SNPs and their position in the corresponding protein sequence are also indicated.

NC = non-coding; ND1, 2, 3, 4, 4L, 5, 6 = NADH dehydrogenase, subunit 1, 2, 3, 4, 4L, 5, 6; COX1, 2, 3 = Cytochrome C oxidase, subunit 1, 2, 3; CytB = Cytochrome B; ATP6 = ATP synthase subunit 6.

SNP analysis was also performed on females from the non-selected P0-generations sampled in 2015 (n = 4 and n = 7 for Ls A and Ls V, respectively) and non-selected females from the same strains collected in 2013 (n = 4 and n = 5 for Ls A and Ls V, respectively). The nucleotide sequences for all the mt-genes from all analysed samples (2013, P0 and F2 parasites) are aligned in the Supplementary_File_Alignment.

All Ls A samples from 2013 and three out of four parasites from the P0 Ls A group were of genotype S. In the Ls V strain, all samples from 2013 and four out of seven parasites in the P0 group were of genotype R. However, one P0 Ls A (LsAP0_3) and one P0 Ls V sample (LsVP0_7) was identified as outliers (see the Gene expression paragraph), most likely due to mislabelling of the samples during collection. Thus, after removing these samples from the results, all Ls A samples were of genotype S and all except two P0 samples in the Ls V strain were of genotype R (LsVP0_2 and LsVP0_5). The latter two samples carried six of the 19 SNPs defining the R genotype.

Heteroplasmy did not seem to affect the phenotypes as the homogeneity of the non-synonymous SNPs in *ND1*, *ND5*, *COX1* and *COX3* within single parasites was more than 97% for all genes, and in most cases above 99% (Supplementary Material, Table S2).

Additional F2 parasites that did not undergo transcriptome sequencing, were analysed by TaqMan assay for the presence of the non-synonymous SNP in *COX1* (*T8605C*), leading to the amino acid substitution Leu107Ser. All parasites characterized as sensitive by bioassay carried the wild type *T8605*, whereas parasites characterized as resistant by bioassay carried the SNP *8605C* (Fig. 1).

### Gene expression analysis

Transcriptome analysis was performed on female parasites from the fully sensitive strain (Ls A) and the pyrethroid resistant strain (Ls V) sampled in 2013, the P0 generation sampled in 2015 and from the individually characterized parasites in the F2-generation. The sequencing depth (mean ± standard deviation) was 18.9 ± 1.9 million reads for the F2 samples, 31.1 ± 4.0 million reads for the P0 samples and 28.4 ± 5.2 million reads for the 2013 samples. On average 3.4 ± 0.6% of the reads aligned to the mitochondrial genes.

**Figure 2:**
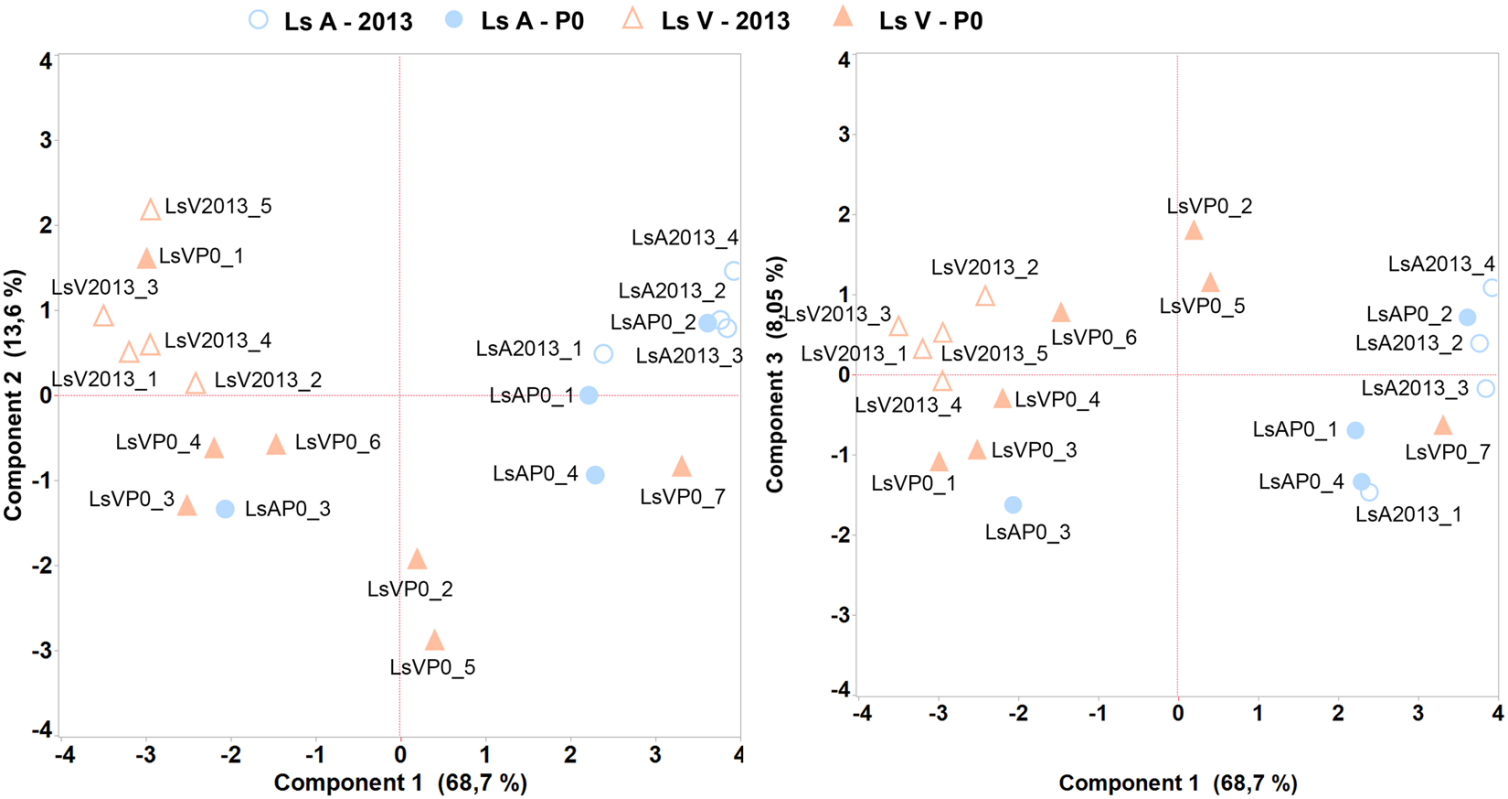
Principal component analysis (PCA) plot of the mitochondrial gene expression. Blue dots represent parasites from the sensitive strain Ls A from 2013 (open circles) and P0-generation (solid circles). Red dots represent parasites from the resistant strain LS V from 2013 (open triangles) and P0-generation (solid triangles).

Based on Transcripts Per Million (TPM) normalized expression levels, Ls A and Ls V parasites were separated in different groups, as shown in a principal component analysis (Fig. 2). However, two of the P0 samples (LsAP0_3 and LsVP0_7) clustered together with the opposite group, indicating that these two samples were outliers. This was supported by their individual non-synonymous SNP profile as explained above, as LsAP0_3 carried all SNPs defining the resistant type (Ls V) and LsVP0_7 carried the wildtypes, as the rest of the Ls A parasites. The presence of a SNP associated with resistance to another compound could distinguish a louse from a fully sensitive strain, such as Ls A, from a louse from a multi-resistant strain, such as Ls V. The suspected outliers were therefore examined for the SNP associated with azamethiphos resistance in *L*. *salmonis ace1a*^19^. LsVP0_7 did not, and LsAP0_3 did carry this SNP (data not shown), supporting that they belong to the opposite groups. Based on the PCA plot, SNP analysis and the absence or presence of the marker for azamethiphos resistance, these two parasites were considered to be outliers, possibly due to mislabelling during sampling and they were excluded from further analysis. Two other samples from the Ls V P0 group (LsVP0_2 and LsVP0_5) clustered between the Ls A and Ls V. Their non-synonymous SNP profile was not the typical Ls A-profile, but rather a separate profile from both Ls A and Ls V. (Supplementary_File_Alignment). Since Ls V was an unselected strain and most likely a mixture of parasite with different sensitivities, these parasites were not excluded.

**Figure 3:**
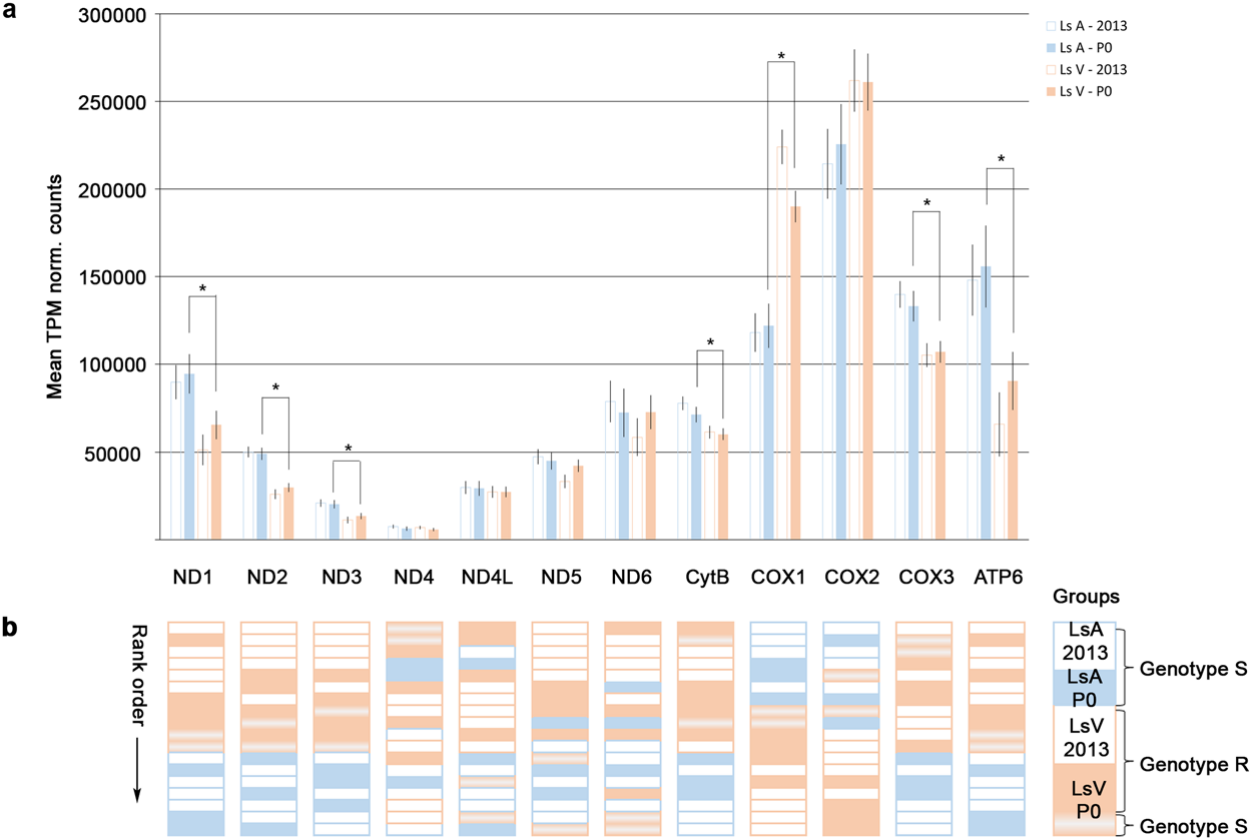
Expression levels of the mitochondrial genes in the 2013 samples and the P0 generation. Transcripts Per Million (TPM) normalized counts are presented as mean ± 95% confidence interval in panel a, with statistically significant differences between strains in the P0 generation indicated by an asterisk (*). The corresponding rank orders of the TPM normalized counts are shown in panel b, with lowest expression at the top. Each block represents one individual. Samples per group: n(Ls A-2013) = 4, n(Ls V-2013) = 5, n(Ls A-P0) = 3, n(Ls V-P0) = 6, n(S-F2) = 5 and n(R-F2) = 5.

TPM normalized expression levels for all mt-genes in adult females from 2013 and the P0 generation are presented in Fig. 3a. For all groups, the most highly expressed gene was *COX2*, followed by *COX1*, *COX3* and *ATP6* with some group-dependent differences in the subsequent order. The lowest expression was found in *ND4*, with approximately 20 times lower expression than *COX2*. Oneway analysis of variance (ANOVA) on the P0 generation revealed a statistically significant effect of strain in seven of the twelve mt-genes, six genes with higher expression in Ls A (*ND1*, *ND2*, *ND3*, *CytB*, *COX3* and *ATP6*) and one gene with higher expression in Ls V (*COX1*). The same result was obtained with non-parametric analysis as illustrated by the rank order of each individual (Fig. 3b). The statistical report is presented as Table S3 in the Supplementary Material.

The 2013 samples were not included in the statistical analysis since Ls V 2013 were field samples and not reared in the lab. Furthermore, the counts from the sensitive and resistant F2 lice could not be directly compared since they were exposed to different concentrations of DMT. The TPM normalized counts from these parasites are presented in Supplementary Material (Table S4).

### 3D models of proteins

The SWISS MODEL gave a GMQE score (a parameter between 0 and 1, where a higher number indicates a better fit) between 0.76 and 0.85 for the final model with the cytochrome C oxidase subunits (COX1-3), and between 0.45 and 0.73 for the NADP dehydrogenase subunits (ND1-6). For the ATP synthase subunit (ATP6), the GMQE score was 0.69.

The 3D models are illustrated in Figures S1–S3 in the Supplementary Material.

### Apoptosis

The first apoptosis experiment was performed on female parasites from two laboratory strains of *Lepeophtheirus salmonis* conferring either DMT resistance (Ls B) or DMT susceptibility (Ls G). The second apoptosis experiment was performed on the same strain conferring DMT resistance (Ls B), but a different strain conferring DMT susceptibility (Ls A, the same strain as used in the crossing study). In the first experiment, parasites were checked hourly to remove dead parasites during the post exposure recovery period. However, no female lice died during this period in any groups. Parasites included in the second experiment were left undisturbed during the recovery period.

**Figure 4:**
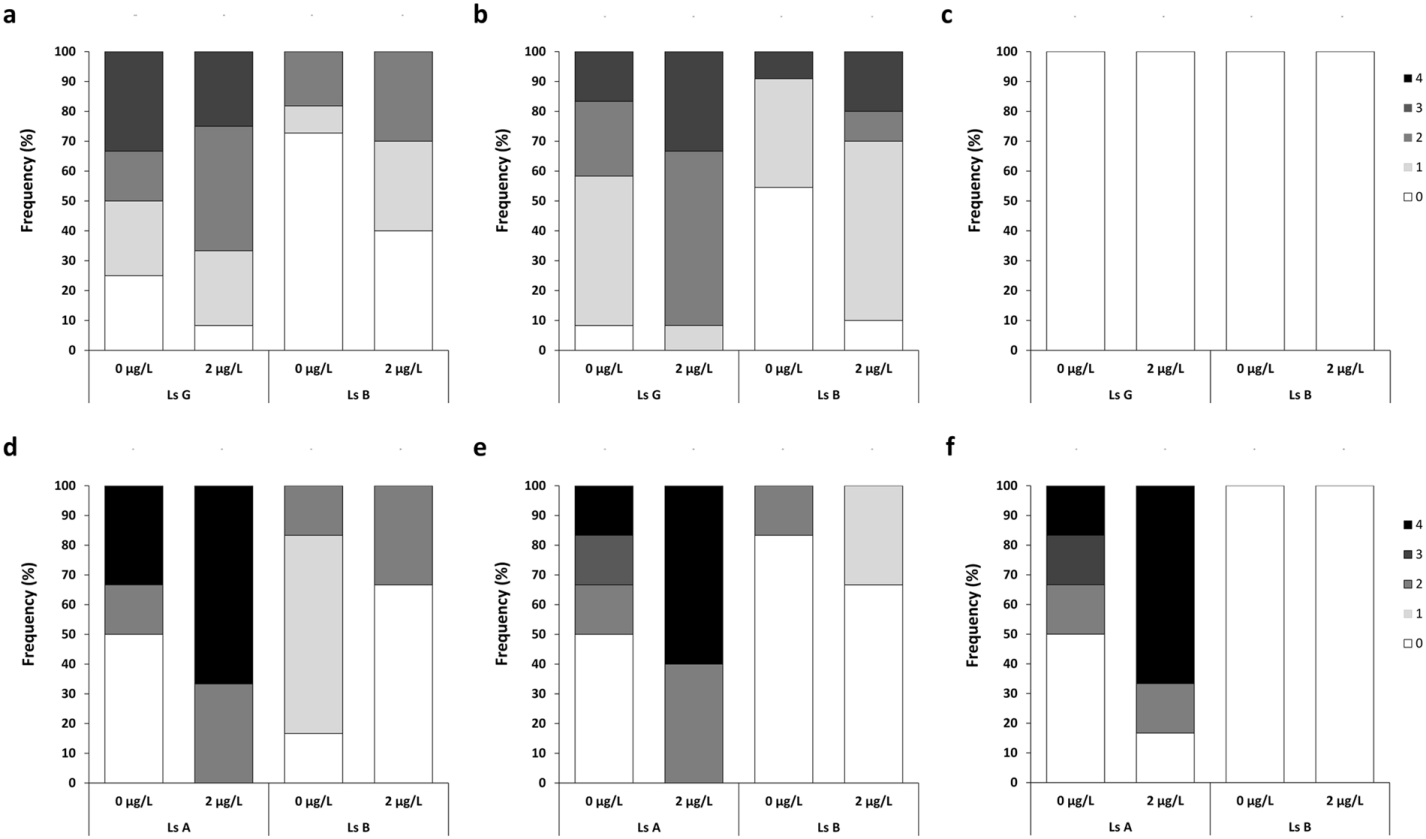
Frequencies of the different levels of apoptosis in subcuticular layer (**a** and **d**), skeletal muscle tissue (**b** and **e**) and central ganglion (**c** and **f**) resulting from experiment 1 (**a**–**c**) or experiment 2 (**d**–**f**). Median values are indicated in brackets. DMT susceptible (Ls G or Ls A) and DMT resistant (Ls B) parasites were exposed to 2 µg DMT/L or vehicle (0 µg/L) for 30 minutes followed by a recovery period of 23.5 hours. In experiment 1, lice were checked regularly throughout the recovery period whereas lice in experiment 2 were left undisturbed. The levels of apoptosis were affected by the handling during the recovery period, as seen as background in vehicle treated lice from both sensitive and resistant strains in experiment 1.

The frequencies of the different apoptotic scores in non-exposed and DMT-exposed parasites are presented in Fig. 4. Ordinal logistic regression analysis was used to verify statistically significant differences.

In skeletal muscle, there was a statistically significant effect of the whole model in both experiments (p = 0.0002 and p = 0.0142 for experiment 1 and 2, respectively). In the first experiment, statistically significant effects of both strain (Ls G and Ls B; p = 0.0049) and dose (0 and 2 µg/L; p = 0.0021) was observed. The level of apoptosis was greater in the susceptible Ls G strain compared to the resistant Ls B strain. Both strains showed an increase in apoptosis after DMT exposure. In the second experiment, statistical effect of strain (Ls A and Ls B) was observed (p = 0.0103) and also the combined effect of strain and DMT concentration was statistically significant (p = 0.0265).

No apoptosis was observed in the nerve tissue in the central ganglion in any of the groups in the first experiment. The apoptotic level in the sensitive Ls A strain included in the second experiment was remarkable, especially after DMT exposure, whereas parasites from the resistant Ls B strain did not show any apoptotic activity.

In the subcuticular layer, the first experiment revealed a statistically significant effect of the whole model (p = 0.0054) and a statistically significant effect of strain on the level of apoptosis (p = 0.0063). The same effect was seen in the second experiment (whole model: p = 0.0033; strain: p = 0.0005). In both experiments, the highest levels of apoptosis were seen in sensitive parasites. No statistically significant effect of the DMT concentration (0 and 2 µg/L) was observed.

## Discussion

The bioassay results on the F2 generation support maternal inheritance of deltamethrin (DMT) resistance in the salmon louse, since resistant individuals, characterized by being not affected after exposure to the high concentration (1 µg/L) of DMT for 24 hours, did not occur in F2 progenies in family group 1, when the mother was from the sensitive strain (Ls A).

As discussed elsewhere^13^, mitochondrial genes rather than chromosomal genes are most likely mediating the maternal inheritance of DMT resistance in the salmon louse. Other explanations for non-mendelian inheritance could be alterations in genes on a female sex chromosome or transfer of symbionts from mother to offspring. A female heterozygous system (ZW/ZZ) has been suggested in the salmon louse^20^, but a genetic marker for resistance on the female W chromosome would exclude resistance development in males. From this study, as well as previously published bioassay results^13,21^, it is evident that males also carry resistance. Thus, maternal inheritance due to alterations on a unique female sex chromosome is less likely. However, the maternal transfer of RNA or protein to the eggs, in which both female and male offspring would receive the maternal effect, cannot be ruled out. Also, resistance transferred by symbiont bacteria^22^ are less likely to be the mediator of maternal inheritance, since DMT resistance appears to be upheld even when the fish, on which the salmon lice feed, are treated with the broad spectrum antibiotic enrofloxacin (personal observation).

The involvement of the mitochondria genes in DMT resistance in the salmon louse has previously been suggested for Norwegian^12^ and Scottish^13^ populations. In terrestrial arthropods such as insects and mites, resistance mechanisms vary in sub-populations of the same species thus resistance strategies cannot be compared (see reviews on e.g. mites^23^, bed bugs^24^ and mosquitos^25^). The North-Atlantic population of salmon lice is genetically very homogeneous^26^, as larvae can be dispersed over a large distance before infecting new fish. In addition, wild salmon from all countries lining the North Atlantic feed on the same feeding grounds north of the Faroe Islands and west of Greenland. Homogeneity has also been demonstrated for the resistance towards the organophosphate azamethiphos, another substance used against sea lice, which is caused by a single mutation in the acetylcholine esterase gene^19^. The mutation is widely dispersed in the North Atlantic population of salmon lice^27^.

Among the 175 SNPs identified in the F2 lice, 20 were non-synonymous (listed in Supplementary Table S1). The bulk of amino acid changes were found in various subunits in the ETC complex I, namely ND1-ND6. In addition, one non-synonymous SNP was located in *ATP6*, and for genes encoding proteins in complex IV, one SNP was located in *COX3* and one in *COX1*, the latter also reported by Nilsen and Espedal^12^ (*T8605C*). Since the mt-genome in general has a high degree of polymorphism, it is unlikely that all these 20 non-synonymous SNPs are involved in DMT-resistance. A study published after the first submission of the current manuscript described differences in the mitochondrial encoded gene sequences between sensitive and resistant *L*. *salmonis* from Scotland^13^. The study was based on progenies from crossing of sensitive and resistant strains, and maternal inheritance was strongly suggested. Four non-synonymous SNPs were identified as unique for resistant parasites from Scotland, identical to SNPs described to cause amino acid substitutions in ND1 (Gly251Ser), ND5 (Leu411Ser), COX1 (Leu107Ser) and COX3 (Gly33Glu) in the current study. As the North-Atlantic population of salmon lice is genetically very homogeneous, the identification of the same resistance-related SNPs in geographically separated strains (from Norway and Scotland) strengthens the assumption that one or more of them are involved in the resistance mechanism, although corroboration from analysis of other strains are needed. Additional SNP analysis of parasites from the P0-generation point in the same direction of an association between the mt-genotype and DMT susceptibility. All individuals from the sensitive Ls A strain shared the same genotype based on the non-synonymous SNPs described in Supplementary Table S1. Furthermore, the parasites from the Ls V strain included both parasites carrying all non-synonymous SNPs and two parasites carrying only six of them(LsVP0_2 and LsVP0_5). Since the Ls V strain was an unselected population of mainly resistant but also susceptible parasites, where the DMT-susceptibility had been determined only on a population level, variation in genotype could be expected. Nevertheless, all F2-parasites that were unaffected by the high DMT concentration (1 µg DMT/L for 24 hours) and analysed by RNAseq, carried all non-synonymous SNPs. This was further supported by the analysis of the *COX1 T8605C* SNP in additional samples from the F2 generation. In family group 1, all males and females shared the wild type allele. In family group 2, all parasites characterised phenotypically as resistance were carrying the SNP, whereas phenotypically sensitive lice all carried the wild type allele. Although the evidence for direct involvement of *COX1* in the DMT resistance is still lacking, the presence of *8605C* in *COX1* indicates that the lice carry a genotype similar to resistant lice whereas the presence of the wildtype *T8605* indicates a genotype similar to sensitive lice. Taken together with the Scottish data^13^, this strongly suggests the involvement of one or more of the Gly251Ser, Leu411Ser, Leu107Ser and Gly33Glu in ND1, ND5, COX1 and COX3, respectively, in the resistance mechanism in *L*. *salmonis*. Influence from the other SNPs detected can though not be ruled out at present.

The differential expression levels of the mt-genes between Ls A and Ls V (P0-generation) were modest (|log2 fold change| < 1) but statistically significant. For *ND1*, *ND2*, *ND3*, *CytB*, *COX3* and *ATP6*, higher expression levels were observed in Ls A compared to Ls V. On the other hand, the expression level of *COX1* was higher in Ls V compared to Ls A, whereas no differences were seen for *ND4*, *ND4L*, *ND5* and *ND6*. The same results were also evident from the rank orders of the individual expression levels for each gene. The two lice in the Ls V-P0 group genotypically separated from the rest of the group (LsVP0_2 and LsVP0_5) were frequently interfering with the rank order of the Ls A parasites, situated between the rest of the Ls V samples and Ls A samples (*ND1*, *COX1* and *ATP6*) or in between the rank order of Ls A samples (*COX2* and *ND5*). It is not known if the gene expression differences could be attributed to the presence or absence of some of the identified SNPs or strain differences in transcription factors or the number of mitochondria in a cell.

To our knowledge, DMT binding to any of the ETC complexes has never been investigated. However, the link between DMT resistance and mitochondrial genes raises the question if such a binding might occur. Therefore, *in silico* docking of DMT to complex I and IV was attempted after 3D modelling of the *L*. *salmonis* mitochondria proteins. Unfortunately, the docking was not conclusive for either complexes. However, one striking observation from the 3D modelling was that the positions of the changed amino acids in COX1 and COX3 (Leu107Ser and Gly33Glu, respectively), were located close to each other with a distance of approximately 9 Å. They are both located on the surface of the protein structure, making them available targets for binding with pyrethroids. If or how the amino acid substitutions observed in resistant parasites could alter the binding properties, if such a binding occurs, is not known. In Complex I, only a few of the amino acid substitutions in ND1-6 were located in the transmembrane domains where DMT binding could be expected. However, they can still have a functional effect on the efficiency of electron transfer or proton translocation and ultimately a change in ATP production.

Increased expression of genes encoding proteins involved in mitochondrial energy metabolism after pyrethroid exposure, including the *ATP synthase*, has been described in *Helicoverpa armigera* midgut^28^. In the water flea, *Daphnia magna*, a susceptible strain displayed a higher energy demand to cope with DMT-induced oxidative stress compared to a resistant strain, and upregulation of proteins involved in apoptosis was mentioned as an effect^29^. A recent study also described differences in the ATP levels between resistant and sensitive salmon lice^13^, adding to the body of evidence that the mitochondria are involved in the resistance mechanism in *L*. *salmonis*. Sensitive male lice were shown to have decreased whole body ATP after DMT exposure (0.2 µg/L for 30 minutes), whereas resistant males upheld the ATP levels after a much higher dose of DMT (2 µg/L for 30 minutes).

Low levels of ATP are known to disrupt the mitochondria membrane potential between the intermembrane space and the cytosol, resulting in leakage of cytochrome C (cytC) into the cytosol which further activates downstream effects such as caspase3 activation and ultimately DNA fragmentation. This is the pathway of controlled cell death, also known as apoptosis. It has previously been shown that DMT can induce apoptosis^15,16^, supporting the findings from the present study. Varying levels of apoptosis were detected in the different tissues, which implies that the levels of apoptosis were correlated to the concentration of DMT in the particular tissue. However, the DMT-distribution to the tissues was not studied. Protection by p-glycoprotein efflux pumps (P-gps) could contribute to the differences observed. Carmona-Antoñanzas *et al*. found 33 genes/transcripts for ATP-binding cassette (ABC) genes^30^, from which 18 could be assigned to ABC subfamilies known to contain drug transporters, in *L*. *salmonis*. Differences in P-gp expression pattern in different strains could also influence the distribution of DMT.

From the apoptotic score derived from the TUNEL stained sections in the first experiment, it is obvious that the experimental handling induced apoptosis in subcuticular layers and in the skeletal muscle in non-exposed and DMT-exposed parasites. It is likely that this observed DNA fragmentation was partially induced by stress as the lice were checked every hour during the post-exposure recovery period. Poley *et al*. showed that cypermethrin induced differential expression in proteins related to the stress signalling pathways of *target of rapamycin* (*TOR*) and *forkhead box protein* (*FOXO*) in *L*. *salmonis*^31^. Stress related genes have also been implied in studies on pyrethroid exposure to other crustaceans^32^, mammals^33^ and fish^34^. It is therefore likely that strains with different sensitivity towards DMT display different responses to stress. To control for the possible bias from handling stress, the level of apoptosis was assessed on TUNEL stained sections from additionally sensitive (Ls A) and resistant (Ls B) lice after DMT or vehicle exposure (experiment 2). When looking at all parasites, stress-induced apoptosis was plausible, as the fraction of non-exposed parasites with apoptosis was decreased from experiment 1 (stressed parasites) to experiment 2 (non-stressed parasites). There were however variations, as evident from Fig. 4. Apoptosis could not be detected in the central ganglion in any groups in experiment 1. In experiment 2 however, the median score increased from 1 to 4 in the sensitive strain whereas the resistant strain remained without displaying apoptosis. The discrepancy between the two sensitive strains could be assigned to differences in DMT distribution based on strain characteristics, allowing DMT to reach the central ganglion in one strain and not the other. The lack of apoptosis in experiment 1 excluded apoptosis in the central ganglion as the main mechanism for DMT toxicity in sea lice although DMT clearly could induced apoptosis, as seen in Ls A in experiment 2.

When the median apoptosis scores in non-DMT exposed and DMT-exposed parasites were compared, an increase was observed in subcuticular layer and muscle in both experiments for the sensitive strains (Ls G and Ls A) and also for the resistant strain in experiment 1. For the resistant strain in experiment 2, the median score was equal (muscle) or lower (subcuticular layer) in exposed group versus non-exposed group. After exposure, high levels of DMT would be expected in tissues close to the cuticle, as described by Sevatdal *et al*. who found high levels of ^14^C-labeled DMT in subcuticular tissues and around the appendices^35^. The high level of DMT-induced apoptosis in skeletal muscle indicates that this tissue is a major target for DMT.

Judging from ours and others micrographs^36^ and the fact that other crustaceans have multinucleated muscle fibres powering their legs during swimming^37^, it is likely that the muscle cells in the salmon lice are multinucleated. Whether individual nuclei in syncytia can undergo apoptosis and selectively eliminate nuclei from the cell is a controversial issue. *In vitro*, apoptotic nuclei have been observed adjacent to normal nuclei^38^, nevertheless it is unclear whether the cells were in the process of dying or survived the apoptotic incidents. In mammals, apoptosis within the intact syncytium is either rare or does not occur at all^39^. One apoptotic nucleus may affect muscle function, most likely sometime after exposure to DMT. If several nuclei inside one single cell is affected, the cell might die, resulting in reduced muscle strength. From the TUNEL stained sections, it was difficult to assess if the nuclei within the muscle tissue were in fact myonuclei or if they could belong to another group of cells, e.g. nerves which are numerous around muscles. Nevertheless, reduced muscle strength after DMT exposure is supported by the personal observation that chalimus stages of *L*. *salmonis* cannot molt after treatment with DMT although they stay alive for several days. Molting is a process that requires muscular strength of the parasite. Furthermore, DMT-induced effects on muscle activity such as swimming behaviour have been widely described in many species. Swimming velocity was impaired in shrimps exposed to DMT^40^, and the equilibrium, coordination and general swimming behaviour of fish larvae and fry were severely affected following exposure to DMT^41,42^. The salmon louse is an ecto-parasite that is stationary on its host. Therefore, a similar effect to altered swimming behaviour would be detachment from the host, which is a profound effect of DMT exposure. These effects could be expected both after neurotoxic effects, i.e. DMT affect sodium currents in nerves, or muscle weakness following mitochondria-induced apoptosis.

Type II pyrethroids, which include DMT, induce a rapid paralysis (within 60 minutes) of exposed insects, known as the knock-down effect^43^. Interestingly, in a controlled treatment test using DMT in therapeutic concentrations on salmon infected with salmon lice, the time at which the probability of 50% detachment of DMT-sensitive parasites was considerably longer, 16.8 hours^44^. This is in contrast to the effect of another neurotoxic compound, azamethiphos, where the corresponding time for azamethiphos-sensitive parasites was 16 minutes. The longer detachment time following DMT-exposure demonstrates that the DMT treatment does not have an immediate knock-down effect on susceptible parasites as seen in other arthropods. Consequently, the effect on signal conduction in nerve cells may not be the primary effect of DMT in salmon lice.

## Conclusion

Results from the current study supports that resistance towards DMT is maternally inherited in the salmon louse. 175 SNPs, of which 20 were non-synonymous and linked to individuals with high tolerance for DMT, were identified. Four of these non-synonymous SNPs have been described on lice geographically separated from the ones included in this study, emphasizing their relevance to DMT resistance. The position of the amino acid changes indicated an involvement in the electron transfer or proton pumping through the respiratory chain in the mitochondria. Finally, DMT induced apoptosis in skeletal muscle tissue, a response that was affected by strain sensitivity towards DMT and directly by DMT exposure, indicating that skeletal muscles are major target tissues for DMT in salmon lice. The results from this study not only provide further insight into the resistance mechanism against DMT in the salmon louse, but also indicates that DMT may have a previously undescribed mode of action that involves binding of DMT (or metabolites) to enzymes in the respiratory chain.

## Methods

All use of fish for salmon lice cultivation was approved by the Norwegian Food Authorities according to the Norwegian Animal Welfare Act (LovData; LOV-2009-06-19-97) and Regulations for the Use of Research Animals (LovData; FOR-2015-06-18-761).

The lice strains used in this study were all laboratory strains reared at either the research facilities of Solbergstrand (The Norwegian Institute for Water Research, NIVA, Drøbak, Norway) for Ls A and Ls V or at the University of Bergen (Bergen, Norway) for Ls G and Ls B, according to the cultivation protocol by Hamre *et al*.^45^. Ls A was a strain originally collected on a fish farm in the Northern part of Norway in 2011. Ls V was collected from a fish farm in Mid-Norway in 2013. The Ls V 2013 samples referred to in the current study were the original field samples of this strain. Ls G has been a lab strain since 2006, when it was collected in the Bergen region. Finally, Ls B originated from a fish farm in Mid-Norway and was collected in 2009.

At the research facility Solbergstrand, lice were removed from fish and transported to the Faculty of Veterinary Medicine, NMBU (University of Life Sciences, Oslo, Norway) prior to experimental treatments. In Bergen, lice were removed from fish at the same place as the experimental treatments were performed. All exposures and subsequent holding times in fresh sea water were done in 1 litre glass bottles held at 10–12 °C with constant aeration.

### Crossing experiment and bioassays

Two well-characterized *L*. *salmonis* strains were used in the study: Ls A (sensitive to all anti-salmon lice chemicals used in Norway, tested by bioassays) and Ls V (resistant to azamethiphos, deltamethrin, emamectin benzoate and hydrogen peroxide, field reports and bioassays). Spot-checks for DMT sensitivity using two concentrations and 24 h exposure were carried out on the strains^46^, confirming reduced sensitivity towards DMT in the Ls V strain and sensitivity in the Ls A strain. Both strains were maintained in continuous cultures without further selection with DMT at the Norwegian Institute for Water Research (NIVA) in Drøbak, Norway, using the cultivation protocol by Hamre *et al*.^45^.

In order to obtain a lice population with a range of DMT sensitivities, a crossing experiment was designed (see Fig. 1). The crossing experiment was largely conducted as described by Nilsen and Espedal^12^. Four Atlantic salmon of approximately 500 g were kept in separate 100 L glass aquaria (one fish per aquarium) supplied with running sea water, salinity 33 parts per thousand at 8 °C. Two fish were infested with approximately 50 Ls A copepodids each and another two fish with the same number of Ls V copepodids to produce the parental generation (P0). All salmon lice were collected from all fish when the lice were in the pre-adult II stage. Then 10 pre-adult II Ls A females and 10 pre-adult II Ls V males from the P0 generation were put back on 2 individual fish (5 on each fish) kept in individual tanks to produce the F1 generation of the family group 1. Two other fish in separate tanks were infested with the same number of Ls A males and Ls V females to produce the F1 generation of family group 2. All P0 lice from both family groups were preserved in RNAlater after removal of the eggstrings and the eggstrings were hatched. The preserved samples were kept at −80 °C. Four fish were infested with copepodids from the F1 generation, two fish with family group 1 and two fish with family group 2. The lice developed to the adult stage, mated, and egg strings for the F2 generation were collected. Approximately 500 copepodids from each of the family groups 1 and 2 (F2) were used for infestation of eight Atlantic salmon for each family group, separated in different tanks.

Some of the preadult F2 lice (mostly preadult II females and young adult males) were removed from the fish under anesthesia (metacaine) and tested for sensitivity towards DMT at 0.2 and 1 µg/L in a 24 h assay^46^. Control groups without DMT were included to check the general performance of the parasites after 24 h under the given experimental conditions. Parasites becoming immobilized at 0.2 µg/L were considered sensitive to DMT, whereas parasites that were not visibly affected at 1 µg/L were considered resistant. The immobilized parasites still had weak movements of limbs. The remaining parasites left on the fish as preadults were allowed to develop to adults and were then selected for sensitivity towards DMT with the same two DMT concentrations and 24 h exposure time. Immediately after the 24 h exposure, immobilized female lice exposed to 0.2 µg/L (n = 15) and unaffected female lice exposed to 1 µg/L (n = 8) were fixed in RNAlater (Sigma) and kept at −80 °C. In addition, 17 sensitive males from family group 1 and two resistant and one sensitive male(s) from family group 2 were also preserved in RNAlater and kept at −80 °C. The immobilization rate was recorded for all parasites. The selections were performed within 6 hours after sampling.

### Transcriptome sequencing

#### RNA extraction

Five adult F2-females immobilized at 0.2 µg/L (S from family group 1) and five adult F2-females not visibly affected at 1 µg/L (R from family group 2) in addition to female parasites from the P0 and 2013 Ls A (n = 4 and n = 3) and Ls V (n = 5 and n = 7) groups were prepared for RNAseq analysis.

Total RNA was extracted from individual adult females using a Trizol protocol combined with RNeasy Mini kit for animal tissues (Qiagen, CA, USA). Lice tissues were disrupted and homogenized in 1 ml Trizol using TissueLyser MM 301 (Qiagen Retsch) and one stainless steel bead of 5 mm diameter (Qiagen). After mixing with 0.2 ml of chloroform and a centrifugation step, the aqueous phase was transferred to a new vial and mixed with one volume of 70% ethanol. Total RNA was then isolated with RNeasy spin columns following manufacturer’s protocol. Genomic DNA was removed from the extracted RNA with Turbo DNA-free TM kit (TURBO™ DNase Treatment and Removal Reagents, Ambion, Life Technologies). Subsequently, the RNA was cleaned and concentrated with RNA Clean & ConcentratorTM-5 (Zymo Research). The RNA was quantified with ND-100 Spectrophotometer (Thermo Fisher Scientific, DE, USA) and the quality was checked with a 2100 Bioanalyzer instrument (Agilent Technologies) and the Agilent RNA 6000 Nano kit.

#### RNAseq

Total RNA samples were used for library preparation and Illumina sequencing at the Norwegian Sequencing Centre (Oslo, Norway). RNAseq libraries (one per individual lice), each with unique index barcodes, were prepared using the TruSeq Stranded total RNA library preparation Kit v2 (Illumina, USA) by following manufacturer’s protocol including the polyA enrichment step. Libraries were pooled together and sequenced on NextSeq500 platform (Illumina, USA) using 150 bp paired end High output reagents. Raw bcl files were generated using RTA v2.4.11 and were later demultiplexed (using sample specific index) and converted to fastq format using bcl2fastq v2.17.1.14. Two separate sequencing runs were performed: one with the F2 parasites and another with the P0 parasites and parasites from 2013.

#### SNP analysis

Nucleotide sequences from each individual parasite were generated for all 12 mitochondria genes using the UGENE tool, version 1.26.1.^47^. This was done by aligning all reads mapping to a specific gene and retrieve the consensus sequence for each individual parasite. To identify SNPs in non-protein coding sequences, alignments to the whole mitochondrial genome were used.

The nucleotide sequences from five F2 resistant and five F2 sensitive parasites undergoing RNAseq analyses were then aligned using MUSCLE^48^. The sequences were translated to amino acids with invertebrate mitochondria genetic codes (Mega version 6^49^) to identify non-synonymous changes. The presence or absence of non-synonymous SNPs was used to define a genotype representing DMT sensitive parasites (genotype S) and a genotype representing DMT resistant parasites (genotype R). The changes defining the genotypes were nineteen of the twenty non-synonymous SNPs listed in Supplementary Table S1. The non-synonymous SNP A7391C, resulting in Met1Ile in ND1, was considered irrelevant for protein function^17^. These nineteen SNPs were also used to characterize the P0 and the 2013 samples that underwent RNAseq analysis. In addition, 25 other parasites from the F2 generation were analysed for one of these SNPs (*COX1 T8605C*) using a TaqMan probe assay (Patogen AS, Ålesund, Norway). The result of the *T8605C* SNP-analysis was used to group the remaining F2 parasites individually characterized as sensitive or resistant by bioassay, but not subjected to RNAseq.

#### Gene expression analysis

Demultiplexed raw reads were cleaned using Trimmomatic v0.33^50^ to remove/trim low quality reads and sequencing adapters as well as using BBMap v34.56 (https://sourceforge.net/projects/bbmap/) to remove reads mapping to PhiX genome (Illumina spike-in). Cleaned fastq reads for each parasite were aligned to the *L*. *salmonis* mitochondrial genome^18^ (GenBank AY625897.1) using Bowtie2 v2.2.6^51^. Reads were aligned both to the 12 individual genes and the complete mitochondrial genome to generate individual bam-files. Unmapped reads were filtered out using Samtools version 1.4^52^. Gene annotation files in GTF format were generated using Cufflinks version 2.2.1.^53^. Counts of fragments aligning to each transcript were calculated using featureCounts version 1.5.2.^54^. On a small data set such as the one in the present study, with only 12 genes, gene-independent normalization offers a more correct presentation of the expression levels of each gene in each sample and therefore also a more truthful representation of differential expression as opposed to other methods such as DESeq2. Therefore, the counts were normalized by the Transcripts Per Million (TPM) method to correct for gene length and sequencing depth^55^. A principal component analysis with robust estimation was performed on the 2013 and P0 samples. Comparison of expression levels between the two strain Ls A and Ls V (P0 samples) was performed with oneway analysis of variance on TPM normalized counts by gene. The significance level was set α = 0.05 (JMP Pro ver. 13.0.0, SAS Institute).

#### 3D modelling of proteins

The three-dimensional (3D) structures of the enzymes were modelled using the Swiss model, ProMod3 Version 1.0.2.^56^. Three models were built to illustrate the relative positions of the identified amino acid changes. In each model, the amino acid sequence for each subunit was fitted to the template separately and the individual subunit models were thereafter combined to a 3D model of all subunits.

The first model was for cytochrome C oxidase (COX) subunits 1, 2 and 3 with cytochrome C (cytC) docked on the enzyme. The model for cytC was generated from the *L*. *salmonis* cytC protein sequence in LiceBase (https://licebase.org/, EMLSAP00000002682) with cytC from equine heart^57^ (PDB id. 1hrc) as template. The model for COX was generated from the *L*. *salmonis* sequences for COX subunits 1, 2 and 3 from sensitive salmon lice (this study) using COX from bovine heart mitochondria^58^ (PDB id. 5iy5), comprising all subunits for this complex, as template. CytC was docked on the COX proteins using the ClusPro 2.0 protein-protein docking service (https://cluspro.bu.edu/login.php?redir=/queue.php).

The second model was for NADH dehydrogenase (ND) in the salmon louse. The model for ND was generated from the *L*. *salmonis* sequences for ND subunits 1, 2, 3, 4, 4L, 5 and 6 from sensitive parasites (this study), using ND from ovine heart mitochondria^59^ (PDB id. 5lnk), comprising all subunits for this complex, as template.

The third model was for ATP-synthase, subunit 6 (ATP6) in the salmon louse. This model was also generated from the *L*. *salmonis* sequences for ATP6 in sensitive parasites (this study) using bovine ATP6^60^ (PDB id. 5ara) as template.

All figures were produced with the Chimera 1.10.2 software software (https://www.cgl.ucsf.edu/chimera/).

### Apoptosis

#### Experimental set up

Adult females from the DMT-sensitive strain Ls G and the DMT-resistant strain Ls B were used for experiment 1 (n = 12 in each group). The DMT sensitivity in Ls G and Ls B, measured as the EC_50_ value 24 h after a 30 min. exposure^61^, were 0.3 µg/L and 39.7 µg/L, respectively. Lice were randomly distributed to the exposure glass bottles filled with 1 litre of sea water within one hour after being removed from the fish. The lice were exposed to 2 µg DMT/L or water (0 µg DMT/L) for 30 minutes whereupon the water was poured out through a mesh and replaced by fresh sea water. Lice that fell onto the mesh were checked for viability and put back into the glass bottle if they could move. No lice were considered dead or immobilized at this point from either strain. The water containing lice was aerated throughout the exposure period and the consecutive 23.5 hours. Four hours after exposure, an hourly inspection of the lice were induced to check for dead lice. To avoid autolysis, dead lice were removed from the bottles whereas only active lice were put back into fresh seawater.

The parasites from Ls A (sensitive strain) and Ls B (resistant strain) included in experiment 2 were subjected to the same protocol as parasites in experiment 1, except that the lice were not checked every hour during the post-exposure period. Instead, the lice were left undisturbed in the period between the refilling of fresh seawater after exposure and the end point (24 h). Due to varying availability of adult lice, parasites included in experiment 2 were not sampled or treated at the same time.

After 24 hours from the start of the exposure, water was poured out of the bottle and onto the mesh. Lice that were stuck to the glass bottles were considered to be unaffected by the exposure. Lice that were collected in the mesh were checked individually for viability. They were characterized as moribund if they were unable to move in sea water and unaffected if they attached to the wall of the test box or could swim in a straight line. Following characterization, the lice were put in 4% buffered paraformaldehyde and fixated for 24–48 hours. Fixated samples were subjected to an automatic paraffin infiltration and subsequently embedded in paraffin wax. The blocks were sectioned on a HM450 microtome (Thermo Fisher) and the sections (5 µm) were collected on Superfrost® glass slides (Thermo Fisher) and kept in the fridge until staining.

#### TUNEL assay

**Figure 5:**
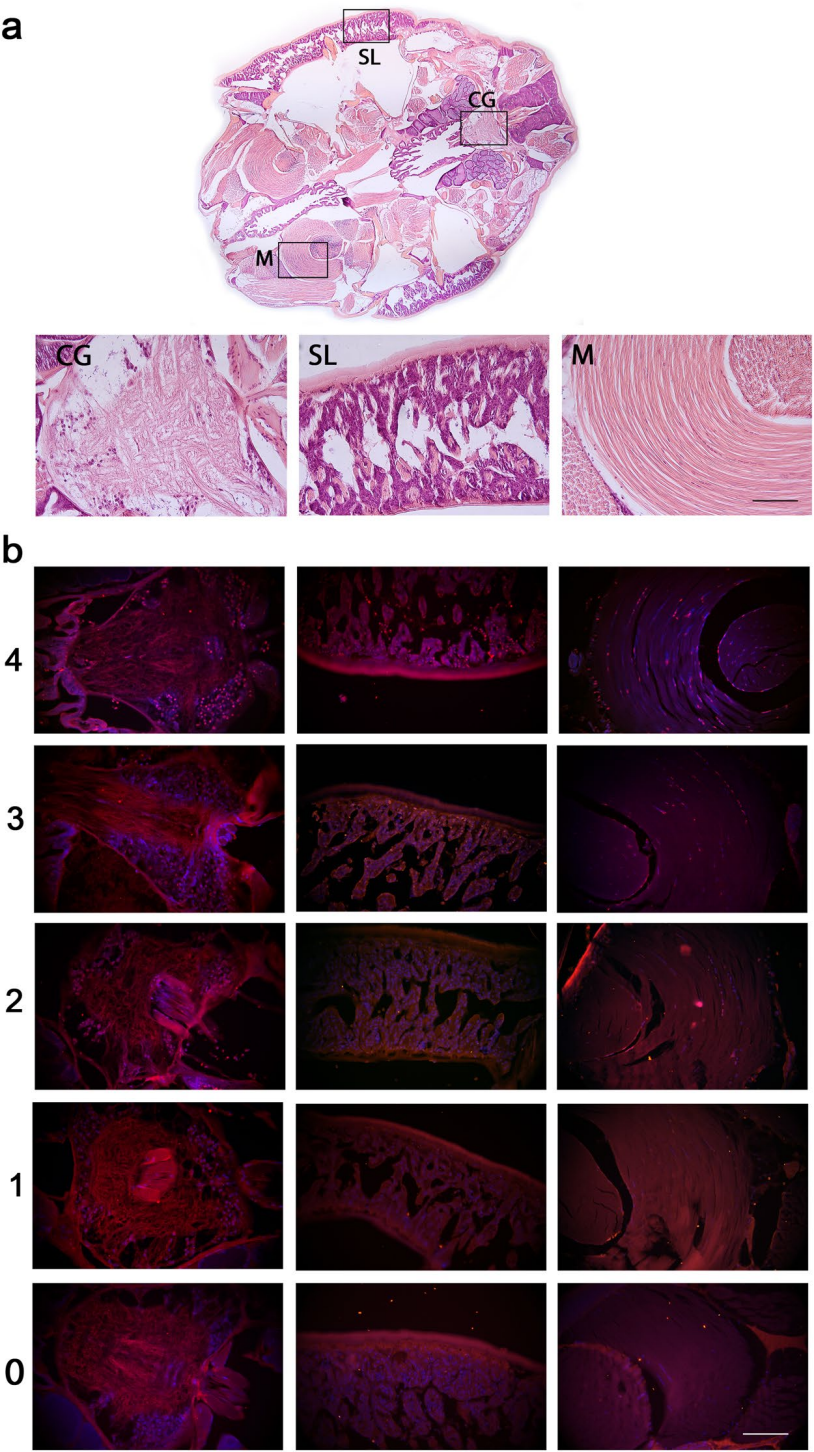
The selected tissues of interest (panel a) in the cephalothorax segment of *L*. *salmonis* were the central ganglion (CG), the subcuticular layer (SL) and skeletal muscle tissue (M). The apoptotic score is shown in panel b. Score 0 (bottom panels) indicates no apoptosis at all, score 4 (top panels) was assigned to the highest levels of apoptosis in each tissue separately. The overview picture in panel a was stained with Haematoxylin and Eosin. The images presented in the apoptotic score panel are overlays of nuclear stain Hoechst (blue) and apoptotic stain TUNEL (yellow). Only spots where Hoechst and TUNEL staining overlap (bright pink) were considered as apoptotic nuclei. The levels of exposure on overlay images were slightly adjusted in Photoshop to enhance to contrast between stained nuclei and the background. Scale bar: 100 µm.

A cell death detection kit from Roche was used to label DNA fragmentation in apoptotic cells (*In Situ* Cell Death Detection Kit, TMR red, Sigma Aldrich, Germany). The protocol that was used followed recommendations from the supplier after dewaxing (60 °C for 30 minutes followed by 3 × 10 minutes in Histoclear®) and rehydration (2 × 1 min in 96% ethanol, 1 × 1 min in 90% ethanol, 1 × 1 min in 70% ethanol and 1 × 1 min in 50% ethanol). The slides were rinsed in PBS (1×) for 1 minute twice whereupon the sections were digested for 5 minutes in a ProteinaseK solution (10 µg/ml) with 10 mM Tris buffer (pH 7.8). In the last washing step after the labelling of DNA strand breaks (the TUNEL stain), the nucleus stain Hoechst (Hoechst 33342, Thermo Fisher Scientific) was added (5 µg/ml) and allowed to work for 15 minutes. An antifade mounting media (SlowFade® Diamond Antifade Mountant with DAPI, Invitrogen^TM^, Thermo Fisher Scientific) was added to the slides before the sections were covered with a cover slip and sealed with nail polish. The slides were investigated under a fluorescent microscope with excitation/emission wavelengths of 530/615 nm and 350/461 nm for TUNEL and Hoechst, respectively. Three areas of interest in the cephalothorax segment (Fig. 5a) were chosen for further analysis; (1) the central ganglion (CG); was chosen based on the high content of nerve cells which is thought to be the primary affected tissue by DMT, (2) the subcuticular layer (SL); was chosen due to its close proximity to the entrance surface for DMT into the parasite, and finally (3) skeletal muscle tissue (M); was chosen due to the high content of mitochondria and the immobilizing effect of DMT. An apoptotic score (Fig. 5b) was made for the subcuticular layer and skeletal muscle tissue, to differentiate between low and high levels of apoptosis. In one end of the scale, the score 0 indicated no apoptosis whereas the score 4 on the opposite end of the scale was assigned to the sections with the highest levels of apoptosis. Scoring of the different tissues was done blind. Frequency of the different apoptotic scores and the interaction of dose and strain was subjected to ordinal logistic regression analysis on SL, M and CG separately, with dose and strain as model effects. The combined effect (strain * dose) was also included in the model. p-values were estimated with effect likelihood ratio test (chi square) with α = 0.05 when the overall model test was statistically significant (α = 0.05). Statistical analysis was performed in JMP Pro 13.0.0 (SAS Institute Inc).

### Data availability

The datasets generated and analysed during the current study are available from the corresponding author on reasonable request.

## Electronic supplementary material

Supplementary_File _Alignment

Supplementary Material
