# Supplementary material for: Deltamethrin resistance in the salmon louse, Lepeophtheirus salmonis (Krøyer): Maternal inheritance and reduced apoptosis
Source: Sci Rep. 2018 May 31;8:8450. doi: 10.1038/s41598-018-26420-6 (PMC5981211; doi:10.1038/s41598-018-26420-6)
Supplement: Supplementary file 2 — Supplementary Material [file 41598_2018_26420_MOESM2_ESM.docx]

**Supplementary Material to “Deltamethrin resistance in the salmon louse, *Lepeophtheirus salmonis* (Krøyer): Maternal inheritance and reduced apoptosis”**

Bakke MJ*, Agusti C, Bruusgaard JC, Sundaram AYM and Horsberg TE

**Content:**

**Page 2 Figure S1:** 3D model of NADH dehydrogenase in the salmon louse.

**Page 3 Figure S2:** 3D model of cytochrome C oxidase with cytochrome C docked on the protein in salmon louse.

**Page 4 Figure S3:** 3D model of ATP synthase subunit 6 in the salmon louse.

**Page 5 Table S1:** A full list of SNPs identified in deltamethrin(DMT)-resistant salmon lice (n=5) compared to sensitive salmon lice (n=5).

**Page 10 Table S2:** The counts of the different nucleotides in each individual parasite.

**Page 13 Table S3:** Statistical report on gene expression analysis.

**Page 24 Table S4:** The transcript per million (TPM) normalized counts for the mitochondria genes in the F2-generation.

**Figure S1:** 3D model of NADH dehydrogenase in the salmon louse. The model for NADH dehydrogenase was generated from the L. salmonis sequences for NADH dehydrogenase subunits 1, 2, 3, 4, 4L, 5 and 6 from sensitive parasites (this study) by the Swiss model (https://swissmodel.expasy.org /) using NADH dehydrogenase from sheep heart (http://www.rcsb.org/pdb/home/home.do PDB id. 5lnk) as template. The figure was produced with the Chimera 1.10.2 software (https://www.cgl.ucsf.edu/chimera/download.html ).

ND3

*M88V*

ND1

*F236L*

ND1

*G251S*

ND3

*T32A*

ND4

*L323M*

ND4

*I371V*

ND6

*T100A*


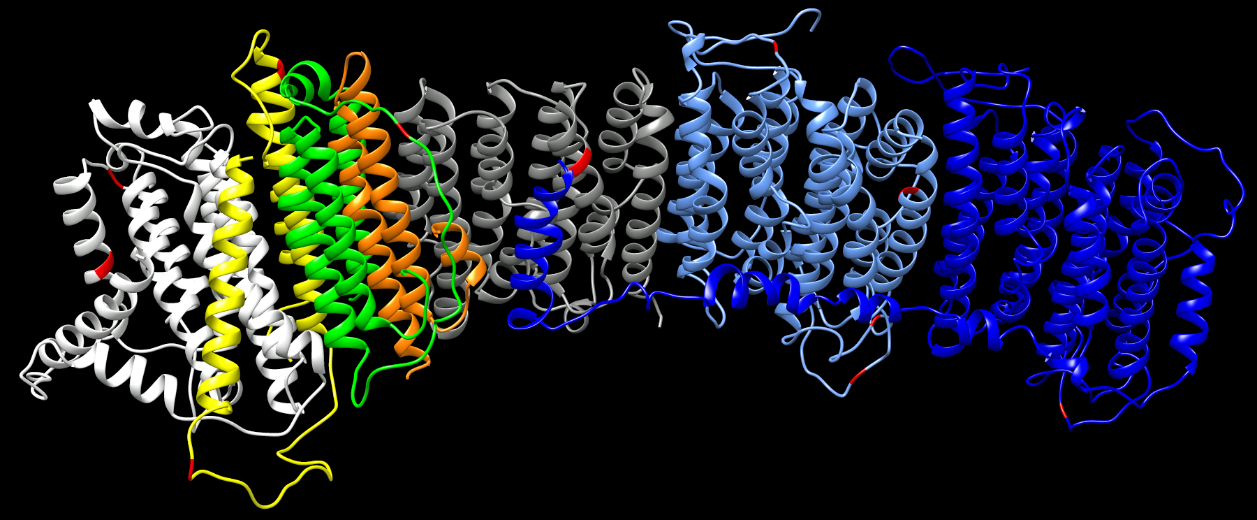


ND2

*G136S*

ND4

*A161T*

ND5

*L411S*

ND4

*S395K*

NADH dehydrogenase subunit 1 (ND1) in white, marked with the site for the changes Phe236Leu and Gly251Ser in red (arrows).NADH dehydrogenase subunit 2 (ND2) in gray, marked with the site for the change Gly136Ser in red (arrow). The changes Ser285Gly, Gly286Ser, Ala296Val and Phe297Leu are not displayed in the model. NADH dehydrogenase subunit 3 (ND3) in yellow, marked with the site for the changes Ala32Gly and Ala88Gly in red (arrows). NADH dehydrogenase subunit 4 (ND4) in light blue, marked with the changes Ala161Thr, Leu323Met, Ile371Val and Ser395Lys in red (arrows).

NADH dehydrogenase subunit 4L (ND4L) in orange. NADH dehydrogenase subunit 5 (ND5) in blue, marked with the change Leu411Ser in red (arrow). NADH dehydrogenase subunit 6 (ND6) in green, marked with the change Thr100Ala in red (arrow). The one-letter abbreviation for amino acids is used in the figure.


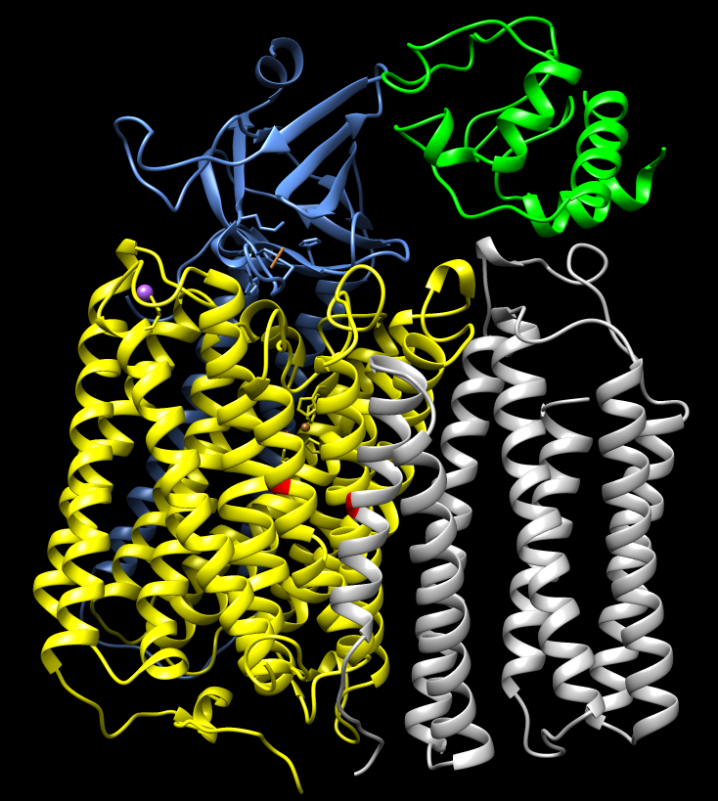


Cu(A)

COx1

*L320S*

Cu(B)

COx3

*G98E*

CytC

**Figure S2:** 3D model of cytochrome C oxidase with cytochrome C docked on the protein in salmon louse. The model for cytochrome C was generated from the L. salmonis cytochrome C protein sequence in LiceBase (https://licebase.org/, EMLSAP00000002682 ) by the Swiss model (https://swissmodel.expasy.org /), with cytochrome C from horse heart (http://www.rcsb.org/pdb/home/home.do, PDB id. 1hrc) as template. The model for cytochrome C oxidase was generated from the L. salmonis sequences for cytochrome C oxidase subunits 1, 2 and 3 from sensitive salmon lice (this study) by the Swiss model using cytochrome C oxidase from cattle heart (PDB id. 5iy5) as template. Cytochrome C was docked on the Cytochrome C oxidase proteins using the ClusPro 2.0 protein-protein docking service (https://cluspro.bu.edu/login.php?redir=/queue.php ). The figure was produced with the Chimera 1.10.2 software (https://www.cgl.ucsf.edu/chimera/download.html ).

Cytochrome C oxidase, subunit 1 (COx1) in yellow, marked with the site for the change Leu320Ser in red (arrow) and the copper-haem binuclear centre (Cu(B), arrow). Cytochrome C oxidase, subunit 2 (COx2) in blue, marked with the binuclear copper binding site (Cu(A), arrow). Cytochrome C oxidase, subunit 3 (COx3) in gray, marked with the site for the change Gly98Glu in red (arrow). Cytochrome C in green.

The one-letter abbreviation for amino acids is used in the figure.


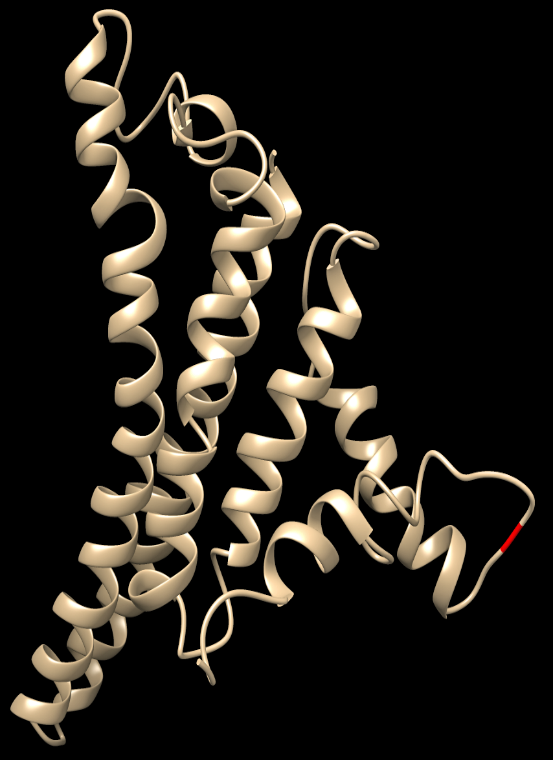


ATP6

*V34A*

**Figure S3:** 3D model of ATP synthase subunit 6 in the salmon louse. The model was generated from the *L. salmonis* sequences for ATP synthase in sensitive parasites (this study) by the Swiss model ([https://swissmodel.expasy.org](https://swissmodel.expasy.org/) /) using ATP synthase from cattle (<http://www.rcsb.org/pdb/home/home.do>PDB id. 5ara) as template. The figure was produced with the Chimera 1.10.2 software (<https://www.cgl.ucsf.edu/chimera/download.html> ).

ATP synthase subunit 6 (ATP6) in gold, marked with the site for the change *Val34Ala* in red (arrow). The one-letter abbreviation for amino acids is used in the figure.

**Table S1:** A full list of SNPs identified in deltamethrin(DMT)-resistant salmon lice (n=5) compared to sensitive salmon lice (n=5). The numbering refers to the mitochondria genome published by Tjensvoll et al., 2005, GenBank AY625897.1. The sequences used for alignment were prepared as consensus sequences from an RNAseq analysis. Salmon lice from a sensitive strain and a resistant strain were crossed, and parasites from the F2 generation were later selected for DMT susceptibility. Sensitive lice were defined as lice becoming moribund after exposure to a low dose of DMT (0.2 µg/L) whereas resistant lice were defined as not visibly affected after exposure to a high dose (1 µg/L) in a 24 h assay. For further details, see text in the material and method section of the manuscript.

| 0 |  | **SNP** | | **Coding triplet** | |  |  | | |
| --- | --- | --- | --- | --- | --- | --- | --- | --- | --- |
| **Position in mt-DNA (†)** | **Gene** | **S** | **R** | **S** | **R** | **Amino acid change** | ***Description*** | | |
| 2 |  | G | S |  |  |  | *Polymorphism* | | |
| 8 |  | C | T |  |  |  | *Polymorphism* | | |
| 114 |  | G | A |  |  |  | *Polymorphism* | | |
| 120 |  | T | G |  |  |  | *Polymorphism* | | |
| 148 |  | T | C |  |  |  | *Polymorphism* | | |
| 174 |  | T | C |  |  |  | *Polymorphism* | | |
| 199 |  | G | A |  |  |  | *Polymorphism* | | |
| 200 |  | A | G |  |  |  | *Polymorphism* | | |
| 204 |  | A | G |  |  |  | *Polymorphism* | | |
| 239 |  | C | T |  |  |  | *Polymorphism* | | |
| 244 |  | G | A |  |  |  | *Polymorphism* | | |
| 254 |  | C | T |  |  |  | *Polymorphism* | | |
| 272 |  | G | A |  |  |  | *Polymorphism* | | |
| 300 |  | A | G |  |  |  | *Polymorphism* | | |
| 304 |  | C | A |  |  |  | *Polymorphism* | | |
| 314 |  | C | T |  |  |  | *Polymorphism* | | |
| 335 |  | G | C |  |  |  | *Polymorphism* | | |
| 373 |  | C | T |  |  |  | *Polymorphism* | | |
| 387 |  | T | C |  |  |  | *Polymorphism* | | |
| 444 |  | G | A |  |  |  | *Polymorphism* | | |
| 521 |  | G | A |  |  |  | *Polymorphism* | | |
| 523 |  | A | G |  |  |  | *Polymorphism* | | |
| 541 |  | C | T |  |  |  | *Polymorphism* | | |
| 551 |  | C | T |  |  |  | *Polymorphism* | | |
| 574 |  | G | A |  |  |  | *Polymorphism* | | |
| 595 |  | C | G |  |  |  | *Polymorphism* | | |
| 598 |  | G | A |  |  |  | *Polymorphism* | | |
| 643 |  | C | T |  |  |  | *Polymorphism* | | |
| 650 |  | T | G |  |  |  | *Polymorphism* | | |
| 656 |  | C | A |  |  |  | *Polymorphism* | | |
| 665 |  | A | G |  |  |  | *Polymorphism* | | |
| 717 |  | A | G |  |  |  | *Polymorphism* | | |
| 726 |  | C | T |  |  |  | *Polymorphism* | | |
| 755 |  | T | C |  |  |  | *Polymorphism* | | |
| 808 |  | G | T |  |  |  | *Polymorphism* | | |
| 851 |  | C | T |  |  |  | *Polymorphism* | | |
| 854 |  | C | T |  |  |  | *Polymorphism* | | |
| 857 |  | G | A |  |  |  | *Polymorphism* | | |
| 887 |  | A | G |  |  |  | *Polymorphism* | | |
| 944 |  | A | G |  |  |  | *Polymorphism* | | |
| 946 |  | G | A |  |  |  | *Polymorphism* | | |
| 948 |  | G | T |  |  |  | *Polymorphism* | | |
| 950 |  | G | A |  |  |  | *Polymorphism* | | |
| 952 |  | C | T |  |  |  | *Polymorphism* | | |
| 1005 | *ND4** | G | C | GTC | GTG | *Val423Val* | *Synonymous* | | |
| **1090** | ***ND4**** | **C** | **T** | **AGA** | **AAA** | ***Ser395Lys*** | ***Non-synonymous*** | | |
| **1163** | ***ND4**** | **T** | **C** | **ATC** | **GTC** | ***Ile371Val*** | ***Non-synonymous*** | | |
| 1179 | *ND4** | G | A | CTC | CTT | *Leu365Leu* | *Synonymous* | | |
| **1307** | ***ND4**** | **A** | **T** | **TTA** | **ATA** | ***Leu323Met*** | ***Non-synonymous*** | | |
| 1398 | *ND4** | C | T | GTG | GTA | *Val292Val* | *Synonymous* | | |
| 1632 | *ND4** | T | C | GGA | GGG | *Gly214Gly* | *Synonymous* | | |
| 1683 | *ND4** | C | T | GAG | GAA | *Glu197Glu* | *Synonymous* | | |
| 1773 | *ND4** | C | G | TCG | TCC | *Ser167Ser* | *Synonymous* | | |
| **1793** | ***ND4**** | **C** | **T** | **GCC** | **ACC** | ***Ala161Thr*** | ***Non-synonymous*** | | |
| 1836 | *ND4** | G | A | ATC | ATT | *Ile146Ile* | *Synonymous* | | |
| 1860 | *ND4** | G | A | GGC | GGT | *Gly138Gly* | *Synonymous* | | |
| 2100 | *ND4** | G | A | GCC | GCT | *Ala58Ala* | *Synonymous* | | |
| 2323 | *ND2* | C | G | GGC | GGG | *Gly10Gly* | *Synonymous* | | |
| 2485 | *ND2* | A | G | AGA | AGG | *Ser64Ser* | *Synonymous* | | |
| 2585 | *ND2* | T | C | TTA | CTA | *Leu98Leu* | *Synonymous* | | |
| **2699** | ***ND2*** | **G** | **A** | **GGT** | **AGT** | ***Gly136Ser*** | ***Non-synonymous*** | | |
| 2732 | *ND2* | T | C | TTA | CTA | *Leu147Leu* | *Synonymous* | | |
| 2824 | *ND2* | C | T | CAC | CAT | *His177His* | *Synonymous* | | |
| 2836 | *ND2* | T | C | GTT | GTC | *Val181Val* | *Synonymous* | | |
| 2842 | *ND2* | T | C | TTT | TTC | *Phe183Phe* | *Synonymous* | | |
| 2845 | *ND2* | T | C | TTT | TTC | *Phe184Phe* | *Synonymous* | | |
| 2878 | *ND2* | G | A | TGG | TGA | *Trp195Trp* | *Synonymous* | | |
| 2935 | *ND2* | G | A | GGG | GGA | *Gly214Gly* | *Synonymous* | | |
| 2962 | *ND2* | G | A | AGG | AGA | *Ser223Ser* | *Synonymous* | | |
| 2992 | *ND2* | C | T | TAC | TAT | *Tyr233Tyr* | *Synonymous* | | |
| 3061 | *ND2* | C | T | CTC | CTT | *Leu256Leu* | *Synonymous* | | |
| **3146** | ***ND2*** | **A** | **G** | **AGG** | **GGG** | ***Ser285Gly*** | ***Non-synonymous*** | | |
| **3149** | ***ND2*** | **G** | **A** | **GGG** | **AGG** | ***Gly286Ser*** | ***Non-synonymous*** | | |
| 3175 | *ND2* | T | C | CCT | CCC | *Pro294Pro* | *Synonymous* | | |
| **3180** | ***ND2*** | **C** | **T** | **GCA** | **GTA** | ***Ala296Val*** | ***Non-synonymous*** | | |
| **3184** | ***ND2*** | **T** | **G** | **TTT** | **TTG** | ***Phe297Leu*** | ***Non-synonymous*** | | |
| 3205 | *ND2* | G | A | TTG | TTA | *Leu304Leu* | *Synonymous* | | |
| 3317 | *COX3* | G | A | TGG | TGA | *Trp24Trp* | *Synonymous* | | |
| **3343** | ***COX3*** | **G** | **A** | **GGG** | **GAG** | ***Gly33Glu*** | ***Non-synonymous*** | | |
| 3353 | *COX3* | T | C | GCT | GCC | *Ala36Ala* | *Synonymous* | | |
| 3422 | *COX3* | C | T | GCC | GCT | *Ala59Ala* | *Synonymous* | | |
| 3464 | *COX3* | T | C | GGT | GGC | *Gly73Gly* | *Synonymous* | | |
| 3575 | *COX3* | T | C | TTT | TTC | *Phe110Phe* | *Synonymous* | | |
| 3632 | *COX3* | A | G | GGA | GGG | *Gly129Gly* | *Synonymous* | | |
| 3641 | *COX3* | T | C | GGT | GGC | *Gly132Gly* | *Synonymous* | | |
| 3845 | *COX3* | A | G | AGA | AGG | *Ser200Ser* | *Synonymous* | | |
| 3920 | *COX3* | A | G | GGA | GGG | *Gly225Gly* | *Synonymous* | | |
| **4404** | ***ND3*** | **A** | **G** | **ACG** | **GCG** | ***Thr32Ala*** | ***Non-synonymous*** | | |
| 4568 | *ND3* | G | A | AGG | AGA | *Ser86Ser* | *Synonymous* | | |
| **4572** | ***ND3*** | **A** | **G** | **ATA** | **GTA** | ***Met88Val*** | ***Non-synonymous*** | | |
| 4617 | *ND3* | T | C | TTA | CTA | *Leu103Leu* | *Synonymous* | | |
| 4622 | *ND3* | C | T | GGC | GGT | *Gly104Gly* | *Synonymous* | | |
| **4745** | ***ND5*** | **G** | **T** | **GGT** | **GTT** | ***Gly28Val*** | ***Non-synonymous*** | | |
| 4792 | *ND5* | C | T | CTA | TTA | *Leu44Leu* | *Synonymous* | | |
| 4884 | *ND5* | A | G | GTA | GTG | *Val74Val* | *Synonymous* | | |
| 4926 | *ND5* | C | T | TAC | TAT | *Tyr88Tyr* | *Synonymous* | | |
| 5037 | *ND5* | G | A | TGG | TGA | *Trp125Trp* | *Synonymous* | | |
| 5043 | *ND5* | C | T | GGC | GGT | *Gly127Gly* | *Synonymous* | | |
| 5070 | *ND5* | T | C | GTT | GTC | *Val136Val* | *Synonymous* | | |
| 5283 | *ND5* | G | A | TTG | TTA | *Leu207Leu* | *Synonymous* | | |
| 5325 | *ND5* | C | T | GTC | GTT | *Val221Val* | *Synonymous* | | |
| 5508 | *ND5* | G | A | GGG | GGA | *Gly282Gly* | *Synonymous* | | |
| 5514 | *ND5* | A | G | ATA | ATG | *Met284Met* | *Synonymous* | | |
| **5894** | ***ND5*** | **T** | **C** | **TTA** | **TCA** | ***Leu411Ser*** | ***Non-synonymous*** | | |
| 5926 | *ND5* | T | C | TTA | CTA | *Leu422Leu* | *Synonymous* | | |
| 5964 | *ND5* | G | A | GGG | GGA | *Gly434Gly* | *Synonymous* | | |
| 5967 | *ND5* | C | T | GGC | GGT | *Gly435Gly* | *Synonymous* | | |
| 6135 | *ND5* | C | T | GAC | GAT | *Asp491Asp* | *Synonymous* | | |
| 6177 | *ND5* | A | G | ATA | ATG | *Met505Met* | *Synonymous* | | |
| 6246 | *ND5* | G | A | GGG | GGA | *Gly528Gly* | *Synonymous* | | |
| 6318 | *ND5* | G | A | GTG | GTA | *Val552Val* | *Synonymous* | | |
| 6330 | *ND5* | G | A | GTG | GTA | *Val556Val* | *Synonymous* | | |
| 6833 | *COX2* | A | G | GGA | GGG | *Gly58Gly* | *Synonymous* | | |
| 6872 | *COX2* | G | A | TTG | TTA | *Leu71Leu* | *Synonymous* | | |
| 6947 | *COX2* | G | A | GGG | GGA | *Gly96Gly* | *Synonymous* | | |
| 6962 | *COX2* | G | A | GGG | GGA | *Gly101Gly* | *Synonymous* | | |
| 6965 | *COX2* | T | C | TAT | TAC | *Tyr102Tyr* | *Synonymous* | | |
| 6980 | *COX2* | C | T | AGC | AGT | *Ser107Ser* | *Synonymous* | | |
| 7166 | *COX2* | G | A | GGG | GGA | *Gly169Gly* | *Synonymous* | | |
| 7307 | *COX2* | T | C | GTT | GTC | *Val216Val* | *Synonymous* | | |
| **7391** | ***ND1*** | **A** | **C** | **ATA** | **ATC** | ***Met1Ile*** | ***Non-synonymous*** | | |
| 7475 | *ND1* | G | A | GGG | GGA | *Gly29Gly* | *Synonymous* | | |
| 7637 | *ND1* | T | C | CCT | CCC | *Pro83Pro* | *Synonymous* | | |
| 7680 | *ND1* | T | C | TTG | CTG | *Leu98Leu* | *Synonymous* | | |
| 8003 | *ND1* | C | T | AAC | AAT | *Asn205Asn* | *Synonymous* | | |
| 8018 | *ND1* | T | C | GTA | GCA | *Phe217Phe* | *Synonymous* | | |
| **8094** | ***ND1*** | **T** | **C** | **TTT** | **CTT** | ***Phe236Leu*** | ***Non-synonymous*** | | |
| **8139** | ***ND1*** | **G** | **A** | **GGG** | **AGG** | ***Gly251Ser*** | ***Non-synonymous*** | | |
| 8426 | *COX1* | G | A | GGG | GGA | *Gly47Gly* | *Synonymous* | | |
| 8558 | *COX1* | C | T | GCC | GCT | *Ala91Ala* | *Synonymous* | | |
| **8605** | ***COX1*** | **T** | **C** | **TTG** | **CTG** | ***Leu107Ser*** | ***Non-synonymous*** | | |
| 8666 | *COX1* | C | T | TAC | TAT | *Tyr127Tyr* | *Synonymous* | | |
| 8759 | *COX1* | A | G | GGA | GGG | *Gly158Gly* | *Synonymous* | | |
| 8849 | *COX1* | C | T | ATC | ATT | *Ile188Ile* | *Synonymous* | | |
| 8954 | *COX1* | A | G | GGA | GGG | *Gly223Gly* | *Synonymous* | | |
| 8963 | *COX1* | C | T | CCC | CCT | *Pro226Pro* | *Synonymous* | | |
| 8972 | *COX1* | T | C | TAT | TAC | *Tyr229Tyr* | *Synonymous* | | |
| 9035 | *COX1* | A | G | GGA | GGG | *Gly250Gly* | *Synonymous* | | |
| 9131 | *COX1* | A | G | GGA | GGG | *Gly82Gly* | *Synonymous* | | |
| 9395 | *COX1* | T | C | TAT | TAC | *Tyr370Tyr* | *Synonymous* | | |
| 9431 | *COX1* | A | G | GGA | GGG | *Gly382Gly* | *Synonymous* | | |
| 9572 | *COX1* | A | G | CTA | CTG | *Leu429Leu* | *Synonymous* | | |
| 9647 | *COX1* | C | T | TTC | TTT | *Phe454Phe* | *Synonymous* | | |
| 9668 | *COX1* | T | C | TTT | TTC | *Phe461Phe* | *Synonymous* | | |
| 10079 | *rRNA* | A | G |  |  |  | *Polymorphism* | | |
| 10099 | *rRNA* | G | A |  |  |  | *Polymorphism* | | |
| 10183 | *rRNA* | A | G |  |  |  | *Polymorphism* | | |
| 10782 | *rRNA* | A | G |  |  |  | *Polymorphism* | | |
| 10821 | *rRNA* | A | G |  |  |  | *Polymorphism* | | |
| 10844 | *tRNA(Ile)* | T | C |  |  |  | *Polymorphism* | | |
| 11193 | *rRNA* | C | T |  |  |  | *Polymorphism* | | |
| 11882 | *ND6* | A | G | TGA | TGG | *Trp88Trp* | *Synonymous* | | |
| 11913 | *ND6* | C | T | CTA | TTA | *Leu99Leu* | *Synonymous* | | |
| **11916** | ***ND6*** | **A** | **G** | **ACT** | **GCT** | ***Thr100Ala*** | ***Non-synonymous*** | | |
| 12308 |  | T | C |  |  |  | *Polymorphism* | | |
| 12352 | *ND4L** | C | T | AAG | AAA | *Lys98Lys* | *Synonymous* | | |
| 12514 | *ND4L** | C | G | AGG | AGC | *Ser44Ser* | *Synonymous* | | |
| 12592 | *ND4L** | G | A | AGC | AGT | *Ser18Ser* | *Synonymous* | | |
| 12606 | *ND4L** | A | G | TTA | CTA | *Leu14Leu* | *Synonymous* | | |
| 12737 |  | C | T |  |  |  | *Polymorphism* | | |
| 13098 | *ATP6* | T | C | TTA | CTA | *Leu27Leu* | *Synonymous* | | |
| **13120** | ***ATP6*** | **T** | **C** | **GTG** | **GCG** | ***Val34Ala*** | ***Non-synonymous*** | | |
| 13302 | *ATP6* | C | T | CTA | TTA | *Leu95Leu* | *Synonymous* | | |
| 13319 | *ATP6* | C | T | CCC | CCT | *Pro100Pro* | *Synonymous* | | |
| 13505 | *ATP6* | A | T | CTA | CTT | *Leu162Leu* | *Synonymous* | | |
| 13956 | *CYTB** | C | T | ATG | ATA | *Met319Met* | *Synonymous* | | |
| 14016 | *CYTB** | A | G | TTT | TTC | *Phe299Phe* | *Synonymous* | | |
| 14064 | *CYTB** | C | T | TTG | TTA | *Leu283Leu* | *Synonymous* | | |
| 14433 | *CYTB** | C | T | TGG | TGA | *Trp160Trp* | *Synonymous* | | |
| 14727 | *CYTB** | A | G | GAT | GAC | *Val62Val* | *Synonymous* | | |
| 14754 | *CYTB** | C | T | GAG | GAA | *Glu53Glu* | *Synonymous* | | |
| 14862 | *CYTB** | A | G | CCT | CCC | *Pro17Pro* | *Synonymous* | | |
| 15041 |  | C | A |  |  |  | *Polymorphism* | | |
| 15211 |  | C | T |  |  |  | *Polymorphism* | | |
| 15375 |  | A | G |  |  |  | *Polymorphism* | | |
|  | | | | | | | | |  |
| * The genes are read in the reverse direction, thus the significant change in triplets are | | | | | | | |  |  |
| complementary to the SNP and the order of appearance of the amino acids is reversed. | | | | | | | |  |  |
|  | | | | | | | |  |  |
| *ND1, 2, 3, 4, 4L, 5, 6* = the genes encoding NADH dehydrogenase, subunit 1, 2, 3, 4, 4L, 5, 6 | | | | | | | |  |  |
| *COX1, 2, 3* = the genes encoding cytochrome C oxidase, subunit 1, 2, 3 | | | | | | | |  |  |
| *CYTB* = the gene encoding cytochrome B | | | | | | | |  |  |
| *ATP6* = the gene encoding ATP synthase subunit 6 | | | | | | | |  |  |

**Table S2:** The counts of the different nucleotides in each individual parasite, at the SNP positons indicated.

| **Group** | **Parasite ID** | **nucleotide** | ***COX1* (*T8605C*)** | ***COX3* (*G3343A*)** | ***ND1* (*G7391A*)** | ***ND5* (*T5894C*)** |
| --- | --- | --- | --- | --- | --- | --- |
| F2-S | LA | A | 2 | 1 | 1 | 1 |
|  |  | C | 1 | 7 | 1 | 1 |
|  |  | G | 0 | 37867 | 5227 | 0 |
|  |  | T | 11381 | 7 | 0 | 7261 |
|  | LB | A | 4 | 2 | 0 | 0 |
|  |  | C | 0 | 6 | 1 | 0 |
|  |  | G | 0 | 50131 | 5151 | 0 |
|  |  | T | 10644 | 19 | 0 | 7639 |
|  | LC | A | 1 | 2 | 1 | 2 |
|  |  | C | 0 | 14 | 0 | 1 |
|  |  | G | 4 | 44187 | 4916 | 1 |
|  |  | T | 11183 | 12 | 0 | 8471 |
|  | LD | A | 0 | 5 | 0 | 2 |
|  |  | C | 1 | 9 | 0 | 0 |
|  |  | G | 0 | 44799 | 5217 | 0 |
|  |  | T | 10781 | 10 | 0 | 7936 |
|  | LE | A | 0 | 1 | 0 | 2 |
|  |  | C | 2 | 15 | 0 | 2 |
|  |  | G | 1 | 41748 | 5466 | 0 |
|  |  | T | 11206 | 14 | 3 | 6906 |
| F2-R | L1 | A | 5 | 48060 | 4412 | 1 |
|  |  | C | 16221 | 0 | 0 | 4111 |
|  |  | G | 1 | 5 | 14 | 0 |
|  |  | T | 10 | 3 | 0 | 0 |
|  | L2 | A | 2 | 39579 | 2916 | 0 |
|  |  | C | 13032 | 0 | 0 | 4134 |
|  |  | G | 2 | 9 | 8 | 0 |
|  |  | T | 7 | 3 | 0 | 4 |
|  | L3 | A | 3 | 51874 | 3470 | 1 |
|  |  | C | 15953 | 2 | 0 | 4165 |
|  |  | G | 2 | 7 | 12 | 1 |
|  |  | T | 5 | 6 | 0 | 2 |
|  | L4 | A | 2 | 41066 | 2999 | 2 |
|  |  | C | 13441 | 0 | 0 | 3642 |
|  |  | G | 2 | 5 | 13 | 0 |
|  |  | T | 14 | 1 | 0 | 1 |
|  | L5 | A | 2 | 71187 | 5561 | 0 |
|  |  | C | 21325 | 0 | 2 | 6798 |
|  |  | G | 2 | 8 | 24 | 4 |
|  |  | T | 20 | 4 | 0 | 5 |
| Ls A - P0 | Ls17A | A | 4 | 26 | 45 | 9 |
|  |  | C | 14 | 36 | 4 | 7 |
|  |  | G | 13 | 6989 | 15900 | 14 |
|  |  | T | 7818 | 144 | 15 | 16336 |
|  | Ls18A | A | 3 | 28 | 53 | 16 |
|  |  | C | 9 | 34 | 12 | 7 |
|  |  | G | 5 | 7061 | 16213 | 13 |
|  |  | T | 7675 | 155 | 22 | 22691 |
|  | Ls19A | A | 20 | 7618 | 6960 | 5 |
|  |  | C | 16699 | 9 | 4 | 5256 |
|  |  | G | 88 | 28 | 37 | 9 |
|  |  | T | 199 | 39 | 2 | 34 |
|  | Ls20A | A | 1 | 25 | 46 | 5 |
|  |  | C | 19 | 34 | 11 | 7 |
|  |  | G | 9 | 8695 | 21056 | 21 |
|  |  | T | 8930 | 126 | 30 | 22005 |
| Ls V - P0 | Ls9H | A | 25 | 7259 | 4915 | 6 |
|  |  | C | 16353 | 10 | 4 | 4416 |
|  |  | G | 63 | 25 | 38 | 13 |
|  |  | T | 209 | 51 | 1 | 41 |
|  | Ls10H | A | 15 | 36 | 62 | 9 |
|  |  | C | 67 | 30 | 7 | 9 |
|  |  | G | 74 | 6261 | 14934 | 21 |
|  |  | T | 12049 | 87 | 31 | 8158 |
|  | Ls11H | A | 22 | 7952 | 7276 | 7 |
|  |  | C | 18291 | 9 | 13 | 5570 |
|  |  | G | 79 | 23 | 39 | 16 |
|  |  | T | 204 | 47 | 1 | 19 |
|  | Ls12H | A | 34 | 7454 | 7153 | 10 |
|  |  | C | 20315 | 11 | 10 | 6263 |
|  |  | G | 101 | 19 | 48 | 25 |
|  |  | T | 242 | 43 | 0 | 64 |
|  | Ls13H | A | 18 | 38 | 48 | 13 |
|  |  | C | 84 | 37 | 12 | 6 |
|  |  | G | 61 | 6801 | 16235 | 29 |
|  |  | T | 12898 | 108 | 21 | 9678 |
|  | Ls15H | A | 27 | 8268 | 8708 | 13 |
|  |  | C | 18679 | 16 | 4 | 6549 |
|  |  | G | 82 | 19 | 55 | 19 |
|  |  | T | 199 | 40 | 3 | 45 |
|  | Ls16H | A | 2 | 24 | 52 | 14 |
|  |  | C | 10 | 52 | 8 | 5 |
|  |  | G | 6 | 8087 | 19131 | 15 |
|  |  | T | 7848 | 150 | 22 | 23252 |
| Ls A - 2013 | Ls5A | A | 4 | 35 | 49 | 5 |
|  |  | C | 9 | 52 | 10 | 15 |
|  |  | G | 12 | 8127 | 18780 | 22 |
|  |  | T | 8946 | 161 | 21 | 19661 |
|  | Ls6A | A | 0 | 24 | 51 | 9 |
|  |  | C | 13 | 35 | 16 | 7 |
|  |  | G | 8 | 8553 | 18358 | 21 |
|  |  | T | 8545 | 160 | 20 | 23166 |
|  | Ls7A | A | 4 | 36 | 55 | 12 |
|  |  | C | 13 | 44 | 11 | 8 |
|  |  | G | 13 | 9583 | 19931 | 22 |
|  |  | T | 9591 | 201 | 31 | 26514 |
|  | Ls8A | A | 4 | 15 | 34 | 4 |
|  |  | C | 4 | 25 | 8 | 5 |
|  |  | G | 4 | 4422 | 10071 | 13 |
|  |  | T | 4642 | 88 | 15 | 13388 |
| Ls V - 2013 | Ls1H | A | 24 | 4733 | 4355 | 6 |
|  |  | C | 14280 | 12 | 7 | 3118 |
|  |  | G | 93 | 27 | 36 | 5 |
|  |  | T | 195 | 43 | 3 | 18 |
|  | Ls2H | A | 11 | 4779 | 4830 | 5 |
|  |  | C | 11787 | 10 | 7 | 3670 |
|  |  | G | 88 | 25 | 41 | 9 |
|  |  | T | 197 | 47 | 0 | 16 |
|  | Ls3H | A | 27 | 5601 | 4858 | 12 |
|  |  | C | 18687 | 13 | 10 | 3864 |
|  |  | G | 113 | 24 | 44 | 10 |
|  |  | T | 286 | 47 | 1 | 33 |
|  | Ls4H | A | 20 | 6415 | 4634 | 4 |
|  |  | C | 15301 | 11 | 5 | 3901 |
|  |  | G | 71 | 36 | 24 | 7 |
|  |  | T | 208 | 44 | 2 | 32 |
|  | Ls14H | A | 24 | 5173 | 4654 | 4 |
|  |  | C | 14956 | 9 | 2 | 3583 |
|  |  | G | 71 | 17 | 37 | 12 |
|  |  | T | 194 | 36 | 2 | 21 |

**Table S3:** Statistical report on gene expression analysis. Comparison of expression levels between the two strain Ls A and Ls V (P0 samples) was performed with oneway analysis of variance on TPM normalized counts by gene. Additional non-parametric analysis has been included (Wilcoxon rank sum). The significance level was set α=0.05 (JMP Pro ver. 13.0.0, SAS Institute).

**Oneway Analysis of TPM By Strain Gene=LS-ATP6**

**Oneway Anova**

**Summary of Fit**

| Rsquare | 0,682303 |
| --- | --- |
| Adj Rsquare | 0,636918 |
| Root Mean Square Error | 23842,47 |
| Mean of Response | 112161,8 |
| Observations (or Sum Wgts) | 9 |

**Analysis of Variance**

| **Source** | **DF** | **Sum of Squares** | **Mean Square** | **F Ratio** | **Prob > F** |
| --- | --- | --- | --- | --- | --- |
| Strain | 1 | 8546038006 | 8,546e+9 | 15,0336 | 0,0061* |
| Error | 7 | 3979243768 | 568463395 |  |  |
| C. Total | 8 | 1,2525e+10 |  |  |  |

**Means for Oneway Anova**

| **Level** | **Number** | **Mean** | **Std Error** | **Lower 95%** | **Upper 95%** |
| --- | --- | --- | --- | --- | --- |
| Ls A | 3 | 155741 | 13765 | 123191 | 188291 |
| Ls V | 6 | 90372 | 9734 | 67356 | 113389 |

Std Error uses a pooled estimate of error variance

**Wilcoxon / Kruskal-Wallis Tests (Rank Sums)**

| **Level** | **Count** | **Score Sum** | **Expected Score** | **Score Mean** | **(Mean-Mean0)/Std0** |
| --- | --- | --- | --- | --- | --- |
| Ls A | 3 | 24,000 | 15,000 | 8,00000 | 2,195 |
| Ls V | 6 | 21,000 | 30,000 | 3,50000 | -2,195 |

**2-Sample Test, Normal Approximation**

| **S** | **Z** | **Prob>\|Z\|** |
| --- | --- | --- |
| 24 | 2,19469 | 0,0282* |

**1-Way Test, ChiSquare Approximation**

| **ChiSquare** | **DF** | **Prob>ChiSq** |
| --- | --- | --- |
| 5,4000 | 1 | 0,0201* |

Small sample sizes. Refer to statistical tables for tests, rather than large-sample approximations.

**Oneway Analysis of TPM By Strain Gene=LS-COX1**

**Oneway Anova**

**Summary of Fit**

| Rsquare | 0,88699 |
| --- | --- |
| Adj Rsquare | 0,870845 |
| Root Mean Square Error | 12993,45 |
| Mean of Response | 167285,9 |
| Observations (or Sum Wgts) | 9 |

**Analysis of Variance**

| **Source** | **DF** | **Sum of Squares** | **Mean Square** | **F Ratio** | **Prob > F** |
| --- | --- | --- | --- | --- | --- |
| Strain | 1 | 9275719407 | 9,2757e+9 | 54,9412 | 0,0001* |
| Error | 7 | 1181808836 | 168829834 |  |  |
| C. Total | 8 | 1,0458e+10 |  |  |  |

**Means for Oneway Anova**

| **Level** | **Number** | **Mean** | **Std Error** | **Lower 95%** | **Upper 95%** |
| --- | --- | --- | --- | --- | --- |
| Ls A | 3 | 121885 | 7501,8 | 104146 | 139624 |
| Ls V | 6 | 189987 | 5304,6 | 177443 | 202530 |

Std Error uses a pooled estimate of error variance

**Wilcoxon / Kruskal-Wallis Tests (Rank Sums)**

| **Level** | **Count** | **Score Sum** | **Expected Score** | **Score Mean** | **(Mean-Mean0)/Std0** |
| --- | --- | --- | --- | --- | --- |
| Ls A | 3 | 6,000 | 15,000 | 2,00000 | -2,195 |
| Ls V | 6 | 39,000 | 30,000 | 6,50000 | 2,195 |

**2-Sample Test, Normal Approximation**

| **S** | **Z** | **Prob>\|Z\|** |
| --- | --- | --- |
| 6 | -2,19469 | 0,0282* |

**1-Way Test, ChiSquare Approximation**

| **ChiSquare** | **DF** | **Prob>ChiSq** |
| --- | --- | --- |
| 5,4000 | 1 | 0,0201* |

Small sample sizes. Refer to statistical tables for tests, rather than large-sample approximations.

**Oneway Analysis of TPM By Strain Gene=LS-COX2**

**Oneway Anova**

**Summary of Fit**

| Rsquare | 0,377915 |
| --- | --- |
| Adj Rsquare | 0,289046 |
| Root Mean Square Error | 24344,89 |
| Mean of Response | 249207,6 |
| Observations (or Sum Wgts) | 9 |

**Analysis of Variance**

| **Source** | **DF** | **Sum of Squares** | **Mean Square** | **F Ratio** | **Prob > F** |
| --- | --- | --- | --- | --- | --- |
| Strain | 1 | 2520334336 | 2,5203e+9 | 4,2525 | 0,0781 |
| Error | 7 | 4148715556 | 592673651 |  |  |
| C. Total | 8 | 6669049892 |  |  |  |

**Means for Oneway Anova**

| **Level** | **Number** | **Mean** | **Std Error** | **Lower 95%** | **Upper 95%** |
| --- | --- | --- | --- | --- | --- |
| Ls A | 3 | 225542 | 14056 | 192306 | 258778 |
| Ls V | 6 | 261041 | 9939 | 237539 | 284542 |

Std Error uses a pooled estimate of error variance

**Wilcoxon / Kruskal-Wallis Tests (Rank Sums)**

| **Level** | **Count** | **Score Sum** | **Expected Score** | **Score Mean** | **(Mean-Mean0)/Std0** |
| --- | --- | --- | --- | --- | --- |
| Ls A | 3 | 9,000 | 15,000 | 3,00000 | -1,420 |
| Ls V | 6 | 36,000 | 30,000 | 6,00000 | 1,420 |

**2-Sample Test, Normal Approximation**

| **S** | **Z** | **Prob>\|Z\|** |
| --- | --- | --- |
| 9 | -1,42009 | 0,1556 |

**1-Way Test, ChiSquare Approximation**

| **ChiSquare** | **DF** | **Prob>ChiSq** |
| --- | --- | --- |
| 2,4000 | 1 | 0,1213 |

Small sample sizes. Refer to statistical tables for tests, rather than large-sample approximations.

**Oneway Analysis of TPM By Strain Gene=LS-COX3**

**Oneway Anova**

**Summary of Fit**

| Rsquare | 0,79048 |
| --- | --- |
| Adj Rsquare | 0,760548 |
| Root Mean Square Error | 7183,664 |
| Mean of Response | 115671,4 |
| Observations (or Sum Wgts) | 9 |

**Analysis of Variance**

| **Source** | **DF** | **Sum of Squares** | **Mean Square** | **F Ratio** | **Prob > F** |
| --- | --- | --- | --- | --- | --- |
| Strain | 1 | 1362872438 | 1,3629e+9 | 26,4097 | 0,0013* |
| Error | 7 | 361235173 | 51605025 |  |  |
| C. Total | 8 | 1724107610 |  |  |  |

**Means for Oneway Anova**

| **Level** | **Number** | **Mean** | **Std Error** | **Lower 95%** | **Upper 95%** |
| --- | --- | --- | --- | --- | --- |
| Ls A | 3 | 133074 | 4147,5 | 123267 | 142882 |
| Ls V | 6 | 106970 | 2932,7 | 100035 | 113905 |

Std Error uses a pooled estimate of error variance

**Wilcoxon / Kruskal-Wallis Tests (Rank Sums)**

| **Level** | **Count** | **Score Sum** | **Expected Score** | **Score Mean** | **(Mean-Mean0)/Std0** |
| --- | --- | --- | --- | --- | --- |
| Ls A | 3 | 24,000 | 15,000 | 8,00000 | 2,195 |
| Ls V | 6 | 21,000 | 30,000 | 3,50000 | -2,195 |

**2-Sample Test, Normal Approximation**

| **S** | **Z** | **Prob>\|Z\|** |
| --- | --- | --- |
| 24 | 2,19469 | 0,0282* |

**1-Way Test, ChiSquare Approximation**

| **ChiSquare** | **DF** | **Prob>ChiSq** |
| --- | --- | --- |
| 5,4000 | 1 | 0,0201* |

Small sample sizes. Refer to statistical tables for tests, rather than large-sample approximations.

**Oneway Analysis of TPM By Strain Gene=LS-CYTB**

**Oneway Anova**

**Summary of Fit**

| Rsquare | 0,760932 |
| --- | --- |
| Adj Rsquare | 0,726779 |
| Root Mean Square Error | 3305,778 |
| Mean of Response | 63820,22 |
| Observations (or Sum Wgts) | 9 |

**Analysis of Variance**

| **Source** | **DF** | **Sum of Squares** | **Mean Square** | **F Ratio** | **Prob > F** |
| --- | --- | --- | --- | --- | --- |
| Strain | 1 | 243483600 | 243483600 | 22,2804 | 0,0022* |
| Error | 7 | 76497183 | 10928169 |  |  |
| C. Total | 8 | 319980784 |  |  |  |

**Means for Oneway Anova**

| **Level** | **Number** | **Mean** | **Std Error** | **Lower 95%** | **Upper 95%** |
| --- | --- | --- | --- | --- | --- |
| Ls A | 3 | 71176,0 | 1908,6 | 66663 | 75689 |
| Ls V | 6 | 60142,3 | 1349,6 | 56951 | 63334 |

Std Error uses a pooled estimate of error variance

**Wilcoxon / Kruskal-Wallis Tests (Rank Sums)**

| **Level** | **Count** | **Score Sum** | **Expected Score** | **Score Mean** | **(Mean-Mean0)/Std0** |
| --- | --- | --- | --- | --- | --- |
| Ls A | 3 | 24,000 | 15,000 | 8,00000 | 2,195 |
| Ls V | 6 | 21,000 | 30,000 | 3,50000 | -2,195 |

**2-Sample Test, Normal Approximation**

| **S** | **Z** | **Prob>\|Z\|** |
| --- | --- | --- |
| 24 | 2,19469 | 0,0282* |

**1-Way Test, ChiSquare Approximation**

| **ChiSquare** | **DF** | **Prob>ChiSq** |
| --- | --- | --- |
| 5,4000 | 1 | 0,0201* |

Small sample sizes. Refer to statistical tables for tests, rather than large-sample approximations.

**Oneway Analysis of TPM By Strain Gene=LS-ND1**

**Oneway Anova**

**Summary of Fit**

| Rsquare | 0,614683 |
| --- | --- |
| Adj Rsquare | 0,559638 |
| Root Mean Square Error | 12297,59 |
| Mean of Response | 75059,44 |
| Observations (or Sum Wgts) | 9 |

**Analysis of Variance**

| **Source** | **DF** | **Sum of Squares** | **Mean Square** | **F Ratio** | **Prob > F** |
| --- | --- | --- | --- | --- | --- |
| Strain | 1 | 1688773472 | 1,6888e+9 | 11,1669 | 0,0124* |
| Error | 7 | 1058614248 | 151230607 |  |  |
| C. Total | 8 | 2747387720 |  |  |  |

**Means for Oneway Anova**

| **Level** | **Number** | **Mean** | **Std Error** | **Lower 95%** | **Upper 95%** |
| --- | --- | --- | --- | --- | --- |
| Ls A | 3 | 94431,7 | 7100,0 | 77643 | 111221 |
| Ls V | 6 | 65373,3 | 5020,5 | 53502 | 77245 |

Std Error uses a pooled estimate of error variance

**Wilcoxon / Kruskal-Wallis Tests (Rank Sums)**

| **Level** | **Count** | **Score Sum** | **Expected Score** | **Score Mean** | **(Mean-Mean0)/Std0** |
| --- | --- | --- | --- | --- | --- |
| Ls A | 3 | 24,000 | 15,000 | 8,00000 | 2,195 |
| Ls V | 6 | 21,000 | 30,000 | 3,50000 | -2,195 |

**2-Sample Test, Normal Approximation**

| **S** | **Z** | **Prob>\|Z\|** |
| --- | --- | --- |
| 24 | 2,19469 | 0,0282* |

**1-Way Test, ChiSquare Approximation**

| **ChiSquare** | **DF** | **Prob>ChiSq** |
| --- | --- | --- |
| 5,4000 | 1 | 0,0201* |

Small sample sizes. Refer to statistical tables for tests, rather than large-sample approximations.

**Oneway Analysis of TPM By Strain Gene=LS-ND2**

**Oneway Anova**

**Summary of Fit**

| Rsquare | 0,925675 |
| --- | --- |
| Adj Rsquare | 0,915058 |
| Root Mean Square Error | 2917,404 |
| Mean of Response | 36236,56 |
| Observations (or Sum Wgts) | 9 |

**Analysis of Variance**

| **Source** | **DF** | **Sum of Squares** | **Mean Square** | **F Ratio** | **Prob > F** |
| --- | --- | --- | --- | --- | --- |
| Strain | 1 | 742023606 | 742023606 | 87,1816 | <,0001* |
| Error | 7 | 59578707 | 8511243,8 |  |  |
| C. Total | 8 | 801602312 |  |  |  |

**Means for Oneway Anova**

| **Level** | **Number** | **Mean** | **Std Error** | **Lower 95%** | **Upper 95%** |
| --- | --- | --- | --- | --- | --- |
| Ls A | 3 | 49077,7 | 1684,4 | 45095 | 53061 |
| Ls V | 6 | 29816,0 | 1191,0 | 27000 | 32632 |

Std Error uses a pooled estimate of error variance

**Wilcoxon / Kruskal-Wallis Tests (Rank Sums)**

| **Level** | **Count** | **Score Sum** | **Expected Score** | **Score Mean** | **(Mean-Mean0)/Std0** |
| --- | --- | --- | --- | --- | --- |
| Ls A | 3 | 24,000 | 15,000 | 8,00000 | 2,195 |
| Ls V | 6 | 21,000 | 30,000 | 3,50000 | -2,195 |

**2-Sample Test, Normal Approximation**

| **S** | **Z** | **Prob>\|Z\|** |
| --- | --- | --- |
| 24 | 2,19469 | 0,0282* |

**1-Way Test, ChiSquare Approximation**

| **ChiSquare** | **DF** | **Prob>ChiSq** |
| --- | --- | --- |
| 5,4000 | 1 | 0,0201* |

Small sample sizes. Refer to statistical tables for tests, rather than large-sample approximations.

**Oneway Analysis of TPM By Strain Gene=LS-ND3**

**Oneway Anova**

**Summary of Fit**

| Rsquare | 0,872909 |
| --- | --- |
| Adj Rsquare | 0,854753 |
| Root Mean Square Error | 1379,501 |
| Mean of Response | 15761,22 |
| Observations (or Sum Wgts) | 9 |

**Analysis of Variance**

| **Source** | **DF** | **Sum of Squares** | **Mean Square** | **F Ratio** | **Prob > F** |
| --- | --- | --- | --- | --- | --- |
| Strain | 1 | 91494374 | 91494374 | 48,0785 | 0,0002* |
| Error | 7 | 13321156 | 1903022,3 |  |  |
| C. Total | 8 | 104815530 |  |  |  |

**Means for Oneway Anova**

| **Level** | **Number** | **Mean** | **Std Error** | **Lower 95%** | **Upper 95%** |
| --- | --- | --- | --- | --- | --- |
| Ls A | 3 | 20270,3 | 796,46 | 18387 | 22154 |
| Ls V | 6 | 13506,7 | 563,18 | 12175 | 14838 |

Std Error uses a pooled estimate of error variance

**Wilcoxon / Kruskal-Wallis Tests (Rank Sums)**

| **Level** | **Count** | **Score Sum** | **Expected Score** | **Score Mean** | **(Mean-Mean0)/Std0** |
| --- | --- | --- | --- | --- | --- |
| Ls A | 3 | 24,000 | 15,000 | 8,00000 | 2,195 |
| Ls V | 6 | 21,000 | 30,000 | 3,50000 | -2,195 |

**2-Sample Test, Normal Approximation**

| **S** | **Z** | **Prob>\|Z\|** |
| --- | --- | --- |
| 24 | 2,19469 | 0,0282* |

**1-Way Test, ChiSquare Approximation**

| **ChiSquare** | **DF** | **Prob>ChiSq** |
| --- | --- | --- |
| 5,4000 | 1 | 0,0201* |

Small sample sizes. Refer to statistical tables for tests, rather than large-sample approximations.

**Oneway Analysis of TPM By Strain Gene=LS-ND4**

**Oneway Anova**

**Summary of Fit**

| Rsquare | 0,097481 |
| --- | --- |
| Adj Rsquare | -0,03145 |
| Root Mean Square Error | 903,2097 |
| Mean of Response | 5987,111 |
| Observations (or Sum Wgts) | 9 |

**Analysis of Variance**

| **Source** | **DF** | **Sum of Squares** | **Mean Square** | **F Ratio** | **Prob > F** |
| --- | --- | --- | --- | --- | --- |
| Strain | 1 | 616790,2 | 616790 | 0,7561 | 0,4134 |
| Error | 7 | 5710514,7 | 815788 |  |  |
| C. Total | 8 | 6327304,9 |  |  |  |

**Means for Oneway Anova**

| **Level** | **Number** | **Mean** | **Std Error** | **Lower 95%** | **Upper 95%** |
| --- | --- | --- | --- | --- | --- |
| Ls A | 3 | 6357,33 | 521,47 | 5124,3 | 7590,4 |
| Ls V | 6 | 5802,00 | 368,73 | 4930,1 | 6673,9 |

Std Error uses a pooled estimate of error variance

**Wilcoxon / Kruskal-Wallis Tests (Rank Sums)**

| **Level** | **Count** | **Score Sum** | **Expected Score** | **Score Mean** | **(Mean-Mean0)/Std0** |
| --- | --- | --- | --- | --- | --- |
| Ls A | 3 | 18,000 | 15,000 | 6,00000 | 0,645 |
| Ls V | 6 | 27,000 | 30,000 | 4,50000 | -0,645 |

**2-Sample Test, Normal Approximation**

| **S** | **Z** | **Prob>\|Z\|** |
| --- | --- | --- |
| 18 | 0,64550 | 0,5186 |

**1-Way Test, ChiSquare Approximation**

| **ChiSquare** | **DF** | **Prob>ChiSq** |
| --- | --- | --- |
| 0,6000 | 1 | 0,4386 |

Small sample sizes. Refer to statistical tables for tests, rather than large-sample approximations.

**Oneway Analysis of TPM By Strain Gene=LS-ND4L**

**Oneway Anova**

**Summary of Fit**

| Rsquare | 0,067952 |
| --- | --- |
| Adj Rsquare | -0,0652 |
| Root Mean Square Error | 3972,789 |
| Mean of Response | 27970,78 |
| Observations (or Sum Wgts) | 9 |

**Analysis of Variance**

| **Source** | **DF** | **Sum of Squares** | **Mean Square** | **F Ratio** | **Prob > F** |
| --- | --- | --- | --- | --- | --- |
| Strain | 1 | 8054760 | 8054760,1 | 0,5103 | 0,4981 |
| Error | 7 | 110481356 | 15783051 |  |  |
| C. Total | 8 | 118536116 |  |  |  |

**Means for Oneway Anova**

| **Level** | **Number** | **Mean** | **Std Error** | **Lower 95%** | **Upper 95%** |
| --- | --- | --- | --- | --- | --- |
| Ls A | 3 | 29308,7 | 2293,7 | 23885 | 34732 |
| Ls V | 6 | 27301,8 | 1621,9 | 23467 | 31137 |

Std Error uses a pooled estimate of error variance

**Wilcoxon / Kruskal-Wallis Tests (Rank Sums)**

| **Level** | **Count** | **Score Sum** | **Expected Score** | **Score Mean** | **(Mean-Mean0)/Std0** |
| --- | --- | --- | --- | --- | --- |
| Ls A | 3 | 18,000 | 15,000 | 6,00000 | 0,645 |
| Ls V | 6 | 27,000 | 30,000 | 4,50000 | -0,645 |

**2-Sample Test, Normal Approximation**

| **S** | **Z** | **Prob>\|Z\|** |
| --- | --- | --- |
| 18 | 0,64550 | 0,5186 |

**1-Way Test, ChiSquare Approximation**

| **ChiSquare** | **DF** | **Prob>ChiSq** |
| --- | --- | --- |
| 0,6000 | 1 | 0,4386 |

Small sample sizes. Refer to statistical tables for tests, rather than large-sample approximations.

**Oneway Analysis of TPM By Strain Gene=LS-ND5**

**Oneway Anova**

**Summary of Fit**

| Rsquare | 0,088437 |
| --- | --- |
| Adj Rsquare | -0,04179 |
| Root Mean Square Error | 4770,469 |
| Mean of Response | 43167,44 |
| Observations (or Sum Wgts) | 9 |

**Analysis of Variance**

| **Source** | **DF** | **Sum of Squares** | **Mean Square** | **F Ratio** | **Prob > F** |
| --- | --- | --- | --- | --- | --- |
| Strain | 1 | 15454947 | 15454947 | 0,6791 | 0,4371 |
| Error | 7 | 159301604 | 22757372 |  |  |
| C. Total | 8 | 174756550 |  |  |  |

**Means for Oneway Anova**

| **Level** | **Number** | **Mean** | **Std Error** | **Lower 95%** | **Upper 95%** |
| --- | --- | --- | --- | --- | --- |
| Ls A | 3 | 45020,7 | 2754,2 | 38508 | 51533 |
| Ls V | 6 | 42240,8 | 1947,5 | 37636 | 46846 |

Std Error uses a pooled estimate of error variance

**Wilcoxon / Kruskal-Wallis Tests (Rank Sums)**

| **Level** | **Count** | **Score Sum** | **Expected Score** | **Score Mean** | **(Mean-Mean0)/Std0** |
| --- | --- | --- | --- | --- | --- |
| Ls A | 3 | 19,000 | 15,000 | 6,33333 | 0,904 |
| Ls V | 6 | 26,000 | 30,000 | 4,33333 | -0,904 |

**2-Sample Test, Normal Approximation**

| **S** | **Z** | **Prob>\|Z\|** |
| --- | --- | --- |
| 19 | 0,90370 | 0,3662 |

**1-Way Test, ChiSquare Approximation**

| **ChiSquare** | **DF** | **Prob>ChiSq** |
| --- | --- | --- |
| 1,0667 | 1 | 0,3017 |

Small sample sizes. Refer to statistical tables for tests, rather than large-sample approximations.

**Oneway Analysis of TPM By Strain Gene=LS-ND6**

**Oneway Anova**

**Summary of Fit**

| Rsquare | 9,413e-5 |
| --- | --- |
| Adj Rsquare | -0,14275 |
| Root Mean Square Error | 13800,12 |
| Mean of Response | 72484,33 |
| Observations (or Sum Wgts) | 9 |

**Analysis of Variance**

| **Source** | **DF** | **Sum of Squares** | **Mean Square** | **F Ratio** | **Prob > F** |
| --- | --- | --- | --- | --- | --- |
| Strain | 1 | 125500,5 | 125500,5 | 0,0007 | 0,9802 |
| Error | 7 | 1333103930 | 190443419 |  |  |
| C. Total | 8 | 1333229430 |  |  |  |

**Means for Oneway Anova**

| **Level** | **Number** | **Mean** | **Std Error** | **Lower 95%** | **Upper 95%** |
| --- | --- | --- | --- | --- | --- |
| Ls A | 3 | 72317,3 | 7967,5 | 53477 | 91157 |
| Ls V | 6 | 72567,8 | 5633,9 | 59246 | 85890 |

Std Error uses a pooled estimate of error variance

**Wilcoxon / Kruskal-Wallis Tests (Rank Sums)**

| **Level** | **Count** | **Score Sum** | **Expected Score** | **Score Mean** | **(Mean-Mean0)/Std0** |
| --- | --- | --- | --- | --- | --- |
| Ls A | 3 | 12,000 | 15,000 | 4,00000 | -0,645 |
| Ls V | 6 | 33,000 | 30,000 | 5,50000 | 0,645 |

**2-Sample Test, Normal Approximation**

| **S** | **Z** | **Prob>\|Z\|** |
| --- | --- | --- |
| 12 | -0,64550 | 0,5186 |

**1-Way Test, ChiSquare Approximation**

| **ChiSquare** | **DF** | **Prob>ChiSq** |
| --- | --- | --- |
| 0,6000 | 1 | 0,4386 |

Small sample sizes. Refer to statistical tables for tests, rather than large-sample approximations.

**Table S4:** The transcript per million (TPM) normalized counts for the mitochondria genes in the F2-generation. Samples LA-LE were characterized as sensitive by bioassay and hold the genotype S (based on the absence of nineteen non-synonymous SNPs) whereas L1-L5 samples were characterized as resistant by bioassay and hold the genotype R (based on the presence of nineteen non-synonymous SNPs).

|  | LA | LB | LC | LD | LE | L1 | L2 | L3 | L4 | L5 |
| --- | --- | --- | --- | --- | --- | --- | --- | --- | --- | --- |
| LS-ND1 | 72357 | 68929 | 67778 | 74813 | 73384 | 57155 | 51296 | 53951 | 52574 | 51217 |
| LS-ND2 | 48931 | 45279 | 55073 | 39899 | 41764 | 37317 | 38017 | 38726 | 38431 | 35618 |
| LS-ND3 | 59526 | 69253 | 70805 | 54942 | 58496 | 46116 | 58198 | 47107 | 48333 | 53687 |
| LS-ND4 | 10042 | 7109 | 9793 | 8978 | 8804 | 6677 | 7630 | 8020 | 7950 | 9047 |
| LS-ND4L | 57601 | 56353 | 55624 | 52612 | 56407 | 47179 | 44562 | 45877 | 46692 | 44471 |
| LS-ND5 | 66136 | 60233 | 71983 | 56050 | 59563 | 40064 | 51641 | 49363 | 46500 | 47088 |
| LS-ND6 | 38398 | 50095 | 38319 | 52148 | 44980 | 43996 | 47595 | 42885 | 42571 | 44850 |
| LS-CYTB | 56859 | 63384 | 64373 | 62797 | 62545 | 51227 | 52005 | 49177 | 50610 | 54157 |
| LS-COX1 | 145416 | 138954 | 135897 | 144096 | 135760 | 171781 | 163512 | 169159 | 173701 | 178048 |
| LS-COX2 | 189018 | 204923 | 187071 | 205455 | 191936 | 275497 | 245702 | 247857 | 253977 | 249087 |
| LS-COX3 | 142837 | 150074 | 155703 | 143684 | 150601 | 149528 | 152981 | 151717 | 151737 | 147990 |
| LS-ATP6 | 108594 | 104227 | 81211 | 115670 | 132985 | 76482 | 89221 | 89433 | 78606 | 70686 |
